# Hmmr^+^ Fibroblasts Facilitate Hair Regeneration by Biomechanical Sensing of Extracellular Matrix Viscoelasticity

**DOI:** 10.34133/research.1430

**Published:** 2026-09-14

**Authors:** Yuchun Tang, Mengyue Wang, Yuanli Ye, Jingwei Jiang, Wenting Huang, Yiping Zhao, Qiaoli Xie, Jinran Lin, Xiao Xiang, Dan Ke, Chunming Xu, Mingxing Lei

**Affiliations:** ^1^ Key Laboratory of Biorheological Science and Technology of the Ministry of Education & 111 Project Laboratory of Biomechanics and Tissue Repair, College of Bioengineering, Chongqing University, Chongqing 400044, China.; ^2^ Department of Dermatology, Huashan Hospital, Shanghai Institute of Dermatology, State Key Laboratory of Molecular Engineering of Polymers, Fudan University, Shanghai 200040, China.; ^3^ University-Town Hospital of Chongqing Medical University, Chongqing 401331, China.; ^4^ School of Basic Medicine, Gannan Medical University, Ganzhou 341000, China.; ^5^ Jiangxi Provincial Key Laboratory of Tissue Engineering, Gannan Medical University, Ganzhou 341000, China.

## Abstract

How fibroblast heterogeneity orchestrates tissue regeneration by remodeling tissue mechanical properties that guide stem cell function is not well understood. Although fibroblasts are increasingly recognized as key regulators of tissue homeostasis, the specific subpopulations and molecular pathways through which they remodel the mechanical niche during hair follicle regeneration are not fully defined. Here, we identify that Hmmr^+^ fibroblasts located beneath the dermal papilla (DP) of the hair follicle promote hair follicle regeneration by secreting extracellular matrix (ECM) components to activate hair follicle stem cells. Single-cell RNA-sequencing and immunostaining mapped spatially distinct fibroblast subpopulations in the dermal microenvironment and showed that regional ECM viscoelastic remodeling occurs prior to hair regeneration and coincides precisely with the emergence of Hmmr^+^ fibroblasts. Functional studies in vivo and in skin organoids demonstrate that Hmmr^+^ fibroblasts act as mechanical sensors that engage DP cells via NCAM1–FGFR1 signaling to stimulate hair regeneration. We propose a tripartite biomechanical module comprising ECM viscoelastic remodeling (effector), Hmmr^+^ fibroblasts (sensor), and DP cells (executor) that cooperatively drives hair regeneration. Together, our work identifies a heterogeneous fibroblast-defined mechanical niche as a central regulator of tissue renewal, highlighting the role of fibroblast diversity in coordinating regeneration and advancing our understanding of the mechano-molecular basis of tissue repair.

## Introduction

Tissue regeneration is orchestrated by a dynamic exchange of biochemical and mechanical instructions between cells and their local microenvironment [1,2]. Within these mechanical cues, the viscoelasticity of the extracellular matrix (ECM), its ability to simultaneously exhibit energy storage as an elastic solid and energy dissipation as a viscous fluid, has emerged as a key physical cue that regulates cellular behaviors such as spreading, proliferation, and invasion [3,4]. Within this biological system, fibroblasts are canonically known to act as the principal architects of the ECM, regulating basement membrane remodeling, synthesis and assembly of matrix components, and mechanical remodeling of the local tissue microenvironment, which collectively dictate regenerative outcomes [5]. Thus, these processes cannot be achieved without the coordinated activity of distinct fibroblast lineages that act together to choreograph this complex spatiotemporal signaling network within regenerating tissues. However, while the biochemical signaling pathways that regulate tissue regeneration have been extensively characterized, the molecular mechanisms by which different fibroblast subtypes sense, interpret, and transmit mechanical information from the ECM during tissue regeneration remain poorly defined.

The mammalian hair follicle represents a unique regenerative system that undergoes lifelong cycles of growth (anagen), degeneration (catagen), and rest (telogen), each requiring the precise synchronization of cellular activities across multiple compartments [6,7]. Notably, the hair follicle stem cell niche exhibits a unique mechanical architecture, wherein stiff and highly contractile bulge stem cells maintain quiescence, while softer, mechanically dynamic hair germ progenitors facilitate activation [8]. Mechanical forces are sensed through an E-cadherin–PIEZO1 axis, triggering calcium signaling that sustains quiescence and reinforces mechanical properties via an NFATC1/AP1 transcriptional network [9]. In addition, microRNA-205 promotes regeneration by targeting cytoskeletal and junctional components to alter tissue mechanics [8]. These transitions are tightly coupled to dynamic remodeling of the dermal ECM, whose composition and mechanical environment fluctuate to allow follicular morphogenesis [10–13]. One key matrix component, type VI collagen (Col VI), has been recently shown to exert dual regulatory effects on Wnt activity and hair regeneration under both developmental and regenerative conditions [14]. In addition to these biochemical and structural roles, the mechanical state of the ECM also influences immune behavior that is critical for tissue regeneration. Mechanical stretching, for instance, promotes macrophage polarization toward a pro-regenerative M2 phenotype, which in turn activates hair follicle stem cells through the release of paracrine factors such as hepatocyte growth factor (HGF) and insulin-like growth factor 1 (IGF-1), thereby amplifying the regenerative response [15]. In line with this, a recent study has demonstrated that sustained intradermal delivery of acidic fibroblast growth factor (aFGF) effectively activates Wnt/β-catenin signaling in dermal fibroblasts and promotes robust hair regrowth, providing direct in vivo evidence that targeting fibroblast-derived growth factor pathways can recapitulate the regenerative outcomes normally instructed by the mechanical microenvironment [16].

Mesenchymal–epithelial interactions (MEIs) initiate the formation of various epithelial organs through specific molecular events [17–19]. MEI plays an indispensable role in modulating digestive epithelial homeostasis [20], prostate development [21], tooth morphogenesis [22], as well as ductal morphogenesis in the kidney [23] and lung [24]. Furthermore, MEI is essential for both the morphogenesis and regeneration of hair follicles [25]. During early follicle morphogenesis, fibroblasts progressively envelop and compress the follicular placode, generating a centripetal contraction force [26]. This spontaneous aggregation and contraction of dermal cells can trigger mechanosensitive activation of β-catenin in adjacent epithelial cells, directly initiating the genetic programs governing hair follicle formation [26]. While dermal fibroblasts have traditionally been known to be heterogeneous, including papillary dermal fibroblasts, reticular dermal fibroblasts, myofibroblasts, dermal adipocytes, dermal papilla (DP) cells, and dermal sheath (DS) cells, each exerting distinct functions, the advent of single-cell-based sequencing technologies has greatly expanded our understanding of their functional diversity and interconnectivity [27]. Recent studies have revealed that dermal fibroblasts are highly heterogeneous in developmental origin, spatial distribution, and signaling state [27,28]. These fibroblasts all exert distinct functions. Yet, how heterogeneous fibroblast subpopulations remodel ECM and reciprocally decode and relay ECM-derived mechano-chemical information to sustain coordinated cyclic processes during hair regeneration remains poorly understood.

In this study, we investigated how a specialized niche of dermal fibroblasts contributes to hair regeneration during the telogen-to-anagen transition. We identified a distinct fibroblast subset (Hmmr^+^ FBs) that emerges specifically during this transition and responds to hyaluronic acid (HA)-driven ECM remodeling via the hyaluronan-mediated motility receptor (HMMR). We further demonstrated that Hmmr^+^ FBs engage in spatially localized crosstalk with DP through the NCAM1–FGFR1 signaling axis, promoting proliferation and enhancing expression of key regenerative factors such as β-catenin and Lef1. Importantly, we found that HA serves a dual function: It modulates ECM viscoelasticity to convey mechanical cues while also facilitating Hmmr^+^ FBs–DP communication via HA-HMMR binding. Together, our study proposes a tripartite conceptual framework for hair regeneration. This model posits that a sequential biomechanical signaling loop, integrating ECM viscoelastic remodeling (effector), Hmmr^+^ FBs (sensor), and DP (executor), is fundamental to activating the regenerative process. This framework not only clarifies the mechanistic basis of hair cycling but also opens new avenues for therapeutic intervention.

## Results

### Spatiotemporal Hmmr^+^ fibroblast subset emergence during telogen-to-anagen transition

**Fig. 1.**
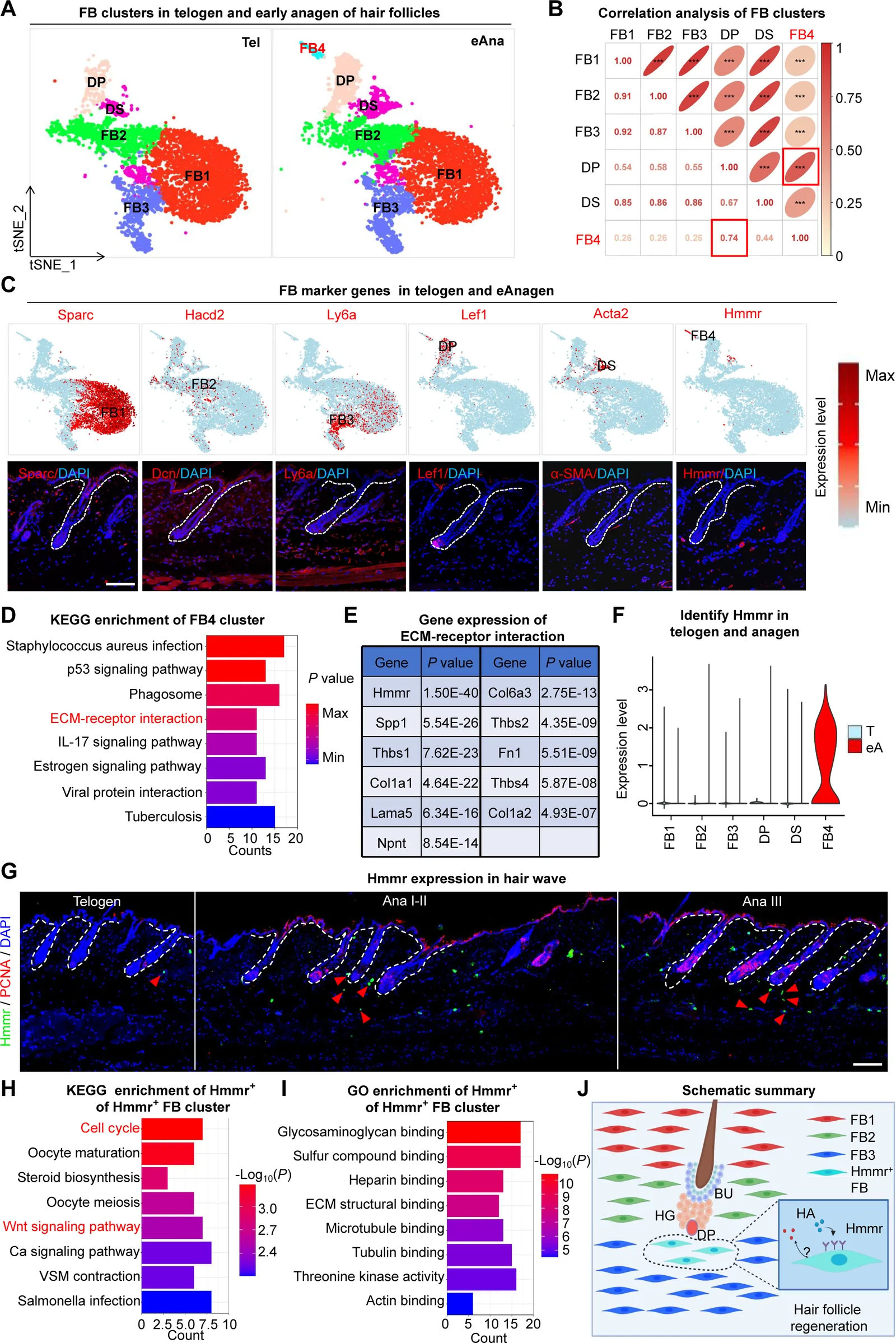
Hmmr^+^ fibroblast subset emergence: Spatiotemporal dynamics in telogen-to-anagen transition. (A) *t*-distributed stochastic neighbor embedding (t-SNE) showing differences in dermal fibroblast subpopulations between telogen and early anagen phases. (B) Correlation analysis of dermal fibroblast subpopulations. (C) Feature plots displaying marker genes for each dermal fibroblast subpopulation, and immunofluorescence (IF) staining illustrating their spatial localization in the skin. Scale bar, 100 μm. (D) KEGG enrichment analysis revealing functional pathways enriched in the FB4 (later referred as “Hmmr^+^ FB). (E) Heatmap of genes involved in ECM–receptor interaction. (F) Violin plot (VlnPlot) demonstrating Hmmr expression in fibroblast subpopulations. (G) IF staining showing Hmmr expression in hair follicle at different stages. Scale bar, 100 μm. (H) Gene Ontology (GO) enrichment analysis highlighting biological functions of genes in Hmmr^+^ FBs. (I) KEGG enrichment analysis highlighting functional roles of genes in Hmmr^+^ FBs. (J) Schematic of the hair follicle microenvironment during early anagen.

To delineate the role of the dermal microenvironment in hair regeneration, we integrated and analyzed our single-cell RNA-sequencing (scRNA-seq) data from mouse skin at telogen (3 weeks) and anagen (4 weeks) stages, with a focused examination of dermal populations (Fig. S1A to C). Reclustering identified a distinct fibroblast subpopulation exclusively present in anagen, which we designated as FB4 (Fig. 1A). Correlation analysis revealed a strong transcriptional similarity between FB4 and DP, implying a potential role for FB4 in shaping the hair follicle niche (Fig. 1B). We next resolved the spatial distribution of these dermal fibroblast subpopulations using immunofluorescence (IF) staining with specific markers. This approach localized FB1 (Sparc^+^/Dcn^+^) to the upper papillary dermis, FB2 (Sparc^−^/Dcn^+^) to the lower papillary dermis, and FB3 (Ly6a^+^) to the reticular dermis. In contrast, FB4 (Hmmr^+^) were specifically enriched within the reticular dermis, directly beneath the DP (Fig. 1C). Functional annotation of FB4 indicated a strong association with hair follicle activity. Kyoto Encyclopedia of Genes and Genomes (KEGG) pathway analysis identified “ECM–receptor interaction” as markedly enriched in this subpopulation (Fig. 1D). Gene Ontology (GO) analysis further linked FB4 to biological processes such as “skin development”, “epithelial cell proliferation”, and “hair cycle”, with corresponding enrichments in cellular components including “collagen-containing ECM” and molecular functions such as “heparin binding” and “glycosaminoglycan binding” (Fig. S1D). These findings suggest that FB4 may actively participate in regulating hair regeneration.

To explore the potential functional role of FB4, we examined genes within the “ECM–receptor interaction” pathway and found that Hmmr was specifically and highly expressed during anagen (Fig. 1E and F and Fig. S1E and F). The up-regulation of Hmmr during the telogen-to-anagen transition was further validated by quantitative reverse transcription polymerase chain reaction (qRT-PCR) and IF staining (Fig. 1G and Fig. S1G). For this reason, we subsequently referred to this anagen-specific fibroblast subpopulation as Hmmr^+^ FBs. Additional enrichment analysis of Hmmr-positive Hmmr^+^ FBs confirmed the association with pathways and GO terms related to hair regeneration (Fig. 1H and I). Collectively, these results identify Hmmr^+^ FBs as a key dermal fibroblast subpopulation specific to the anagen phase and suggest that it may facilitate hair regeneration via an ECM–receptor interaction network centered on Hmmr (Fig. 1J). In addition, as a motility receptor for HA, we hypothesize that Hmmr may exert its function as a mechanosensor.

### HA-Hmmr signaling axis promotes hair regeneration

**Fig. 2.**
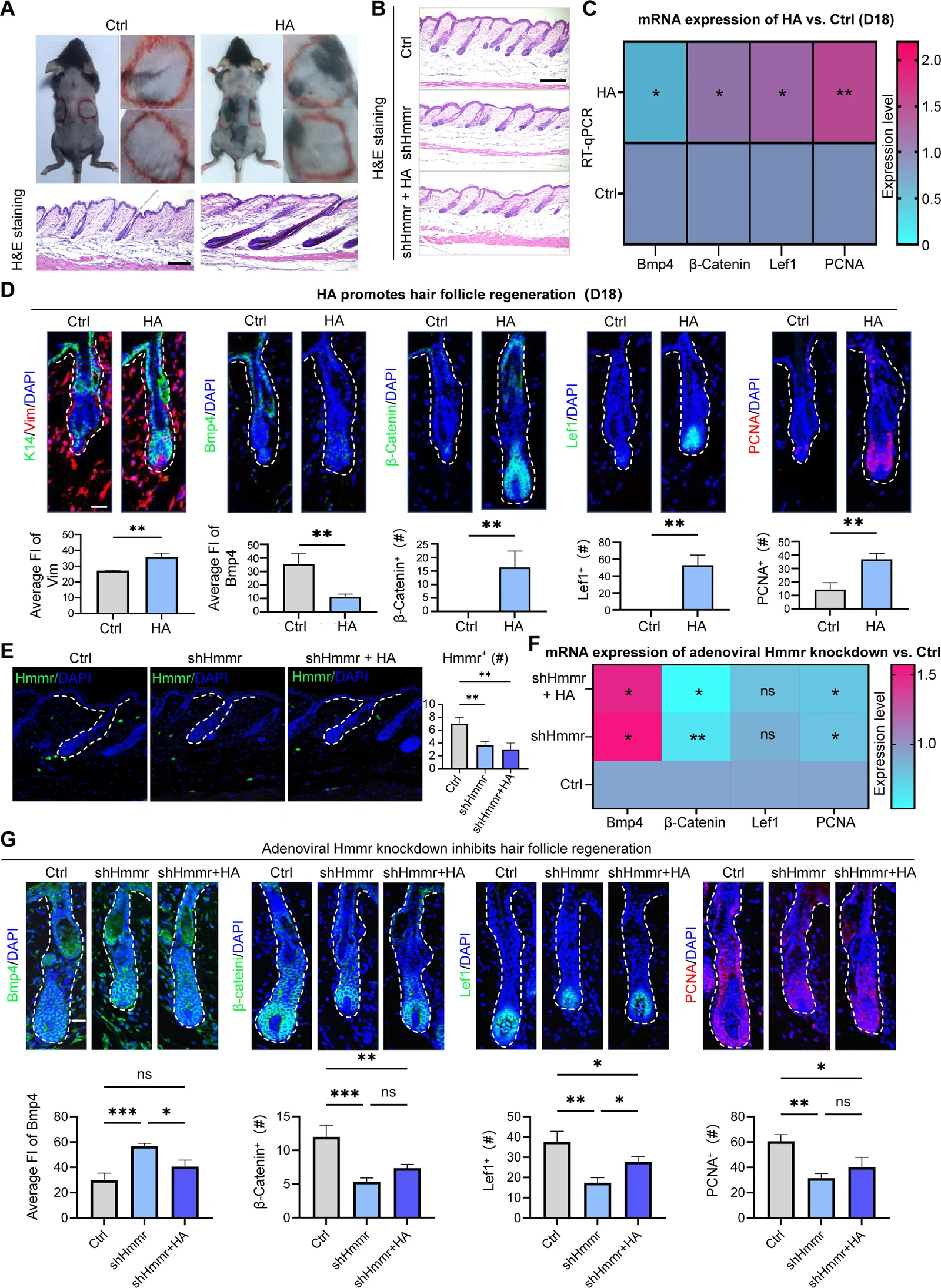
HA-Hmmr signaling drives hair regeneration. (A) Gross photographs and hematoxylin and eosin (H&E) staining images demonstrating the effect of subcutaneous HA injection on hair regeneration. Scale bar, 100 μm. (B) Representative images of H&E staining demonstrating the effect of Hmmr knockdown on hair regeneration. Scale bar, 100 μm. (C) qRT-PCR results showing the effect of HA treatment on the expression of Bmp4, β-catenin, Lef1, and PCNA. (D) IF staining showing the effects of HA treatment on the expression of Bmp4, β-catenin, Lef1, and PCNA on day 18 (D18). Scale bar, 25 μm. (E) IF staining showing Hmmr expression following subcutaneous injection of adenovirus for Hmmr knockdown. (F) qRT-PCR analysis showing the expression of Bmp4, β-catenin, Lef1, and PCNA following subcutaneous adenoviral-mediated Hmmr knockdown. (G) IF staining showing the expression of Bmp4, β-catenin, Lef1, and PCNA following Hmmr knockdown. Scale bar, 25 μm. Mean ± SEM (*n* = 3). Unpaired 2-tailed *t* test or one-way ANOVA with Tukey’s post hoc test. *P < 0.05, **P < 0.01, ***P < 0.001.

As a receptor for HA, HMMR transduces HA-mediated signals into intracellular responses that regulate critical cellular processes such as adhesion, migration, and cell cycle progression [29,30]. To systematically evaluate the role of HMMR in hair regeneration, we employed a dual-model approach. In depilation-induced hair cycling, daily subcutaneous injection of HA accelerated the transition into anagen, as evidenced by histological analysis showing earlier follicle activation compared to the controls (Fig. 2A). Conversely, in the hair plucking model that induces hair regeneration, pharmacological inhibition of HA synthesis using 4-methylumbelliferone (4-MU), or treatment with triptolide (a compound previously reported to suppress Hmmr expression [31]), as well as short hairpin RNA (shRNA)-mediated Hmmr knockdown, greatly impeded regeneration, resulting in notably shorter hair follicles at post-plucking days 4 and 6 (PPD4 and PPD6), as confirmed by hematoxylin and eosin (H&E) and IF staining (Fig. 2A and B and Fig. S2A and B). These complementary results strongly implicate the HA-HMMR axis in the efficient initiation of anagen.

Consistent with this, we performed bulk RNA-seq analysis of 4-MU-treated skin that revealed down-regulation of genes associated with regeneration-related processes, including “hair cycle process”, “epithelial cell differentiation”, and “hair follicle development” (Fig. S2C and D). To assess the involvement of these alterations in follicle regeneration, we next examined related canonical pathway markers. HA treatment reduced Bmp4/BMP4 expression, a signal that maintains telogen [32,33], while increasing Ctnnb1/β-catenin and Lef1/LEF1 (transcription factors involved in follicle development and regeneration [34,35] together with Pcna/PCNA) (Fig. 2C and D).

Prior to functional verification, we validated the efficacy of adenoviral infection and knockdown efficiency by subcutaneous injection in skin tissues. IF staining of green fluorescent protein (GFP), a marker protein carried with adenoviral vector, confirmed successful infection in perifollicular tissues (Fig. S2E). Furthermore, qRT-PCR and IF staining verified that adenoviral delivery of Hmmr-targeting shRNA markedly down-regulated endogenous HMMR expression, compared to the control shRNA (Fig. 2E and Fig. S2F).

In contrast, 4-MU treatment, triptolide intervention, and shRNA-mediated Hmmr knockdown reversed this expression pattern as validated by both qRT-PCR and IF staining (Fig. 2F and G and Fig. S2G to I). Collectively, these findings demonstrate that the HA-HMMR axis promotes hair regeneration through a coordinated mechanism involving suppression of Bmp signaling, activation of Wnt signaling, and enhancement of follicular proliferation.

### An HMMR-high dermal niche sustains hair follicle neogenesis in skin organoids

**Fig. 3.**
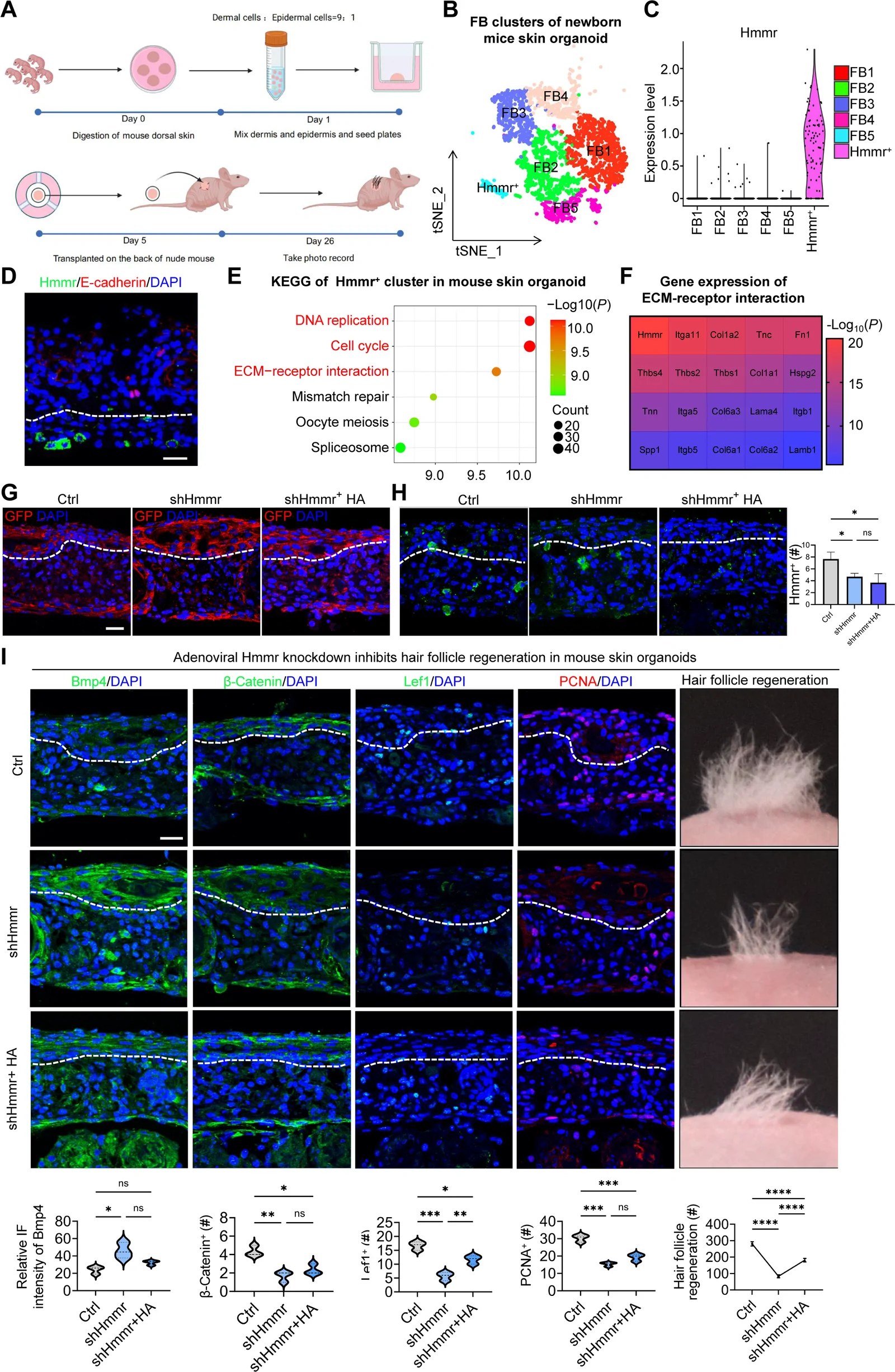
Functional HMMR-high dermal niche enables hair follicle neogenesis in organoids. (A) Schematic of organoid extraction and transplantation experiments in mouse. (B) t-SNE visualization of dermal cell subpopulations in single-cell data from mouse skin organoid. (C) VlnPlot illustrating Hmmr expression in the dermis of mouse skin organoids. (D) IF staining demonstrating Hmmr expression in mouse skin organoid. Scale bar, 25 μm. (E) KEGG enrichment analysis revealing functional pathways in Hmmr^+^ cells. (F) Expression of genes involved in ECM–receptor interaction. (G) IF staining showing GFP expression in mouse hair organoids following adenoviral infection. Scale bar, 25 μm. (H) IF staining showing GFP expression in mouse hair organoids following Hmmr knockdown. Scale bar, 25 μm. (I) Left: IF staining showing the effects of Hmmr knockdown on the expression of Bmp4, β-catenin, Lef1, and PCNA in the mouse skin organoids. Right: Quantitative comparison of hair regeneration after transplantation into nude mice between the knockdown group and the control group. Scale bar, 25 μm. Mean ± SEM (*n* = 3). One-way ANOVA with Tukey’s post hoc test. *P < 0.05, **P < 0.01, ***P < 0.001.

Skin organoids represent a powerful 3-dimensional in vitro model that recapitulates the architectural and functional complexity of native skin, offering a physiologically relevant platform for mechanistic investigation [36]. To further investigate the role of HMMR in hair regeneration, we employed our previously established mouse skin organoid model (Fig. 3A) [13,25,37,38]. Clustering of scRNA-seq data resolved 14 distinct cell subpopulations (Fig. S3A and B), with Hmmr being highly and specifically expressed in a single dermal subset in skin organoids (Fig. 3B and C and Fig. S3C). We accordingly defined this group as Hmmr^+^ dermal cells. IF staining confirmed their predominant localization within the organoid reticular dermis (Fig. 3D), suggesting a potential role in regulating dermal–epidermal crosstalk. KEGG pathway enrichment analysis indicated that Hmmr^+^ cells were remarkably associated with “DNA replication”, “cell cycle”, and “ECM–receptor interaction” (Fig. 3E). Moreover, their expression profile of ECM-related genes closely reflected the stage-specific remodeling events that occur during skin organoid self-assembly (Fig. 3F).

To functionally assess their contribution, we utilized an organoid transplantation model and performed shRNA-mediated Hmmr knockdown in hair organoids, combined with 4-MU and triptolide pharmacological intervention. We first verified the transfection efficiency and knockdown efficacy in vitro. GFP IF staining confirmed successful adenoviral transduction in hair organoids (Fig. 3G). Moreover, IF staining validated that Hmmr shRNA effectively down-regulated its expression in organoids (Fig. 3F). Consistent with the in vivo results, Hmmr knockdown, as well as treatment with 4-MU or triptolide, severely disrupted organoid morphology (Fig. S3D) and considerably reduced the number of regenerated hair follicles (Fig. 3I and Fig. S3E). Mechanistically, HMMR inhibition via pharmacological blockade or shRNA knockdown elevated BMP4 expression while concurrently suppressing the Wnt/β-catenin pathway effectors β-catenin and LEF1 (Fig. 3I and Fig. S3E). Collectively, these organoid-based findings substantiate the essential role of HMMR in hair regeneration, demonstrating that it functions through the modulation of ECM signaling networks and the Wnt/β-catenin pathway.

### Fibroblast-ECM mechanical circuit guides hair regeneration

**Fig. 4.**
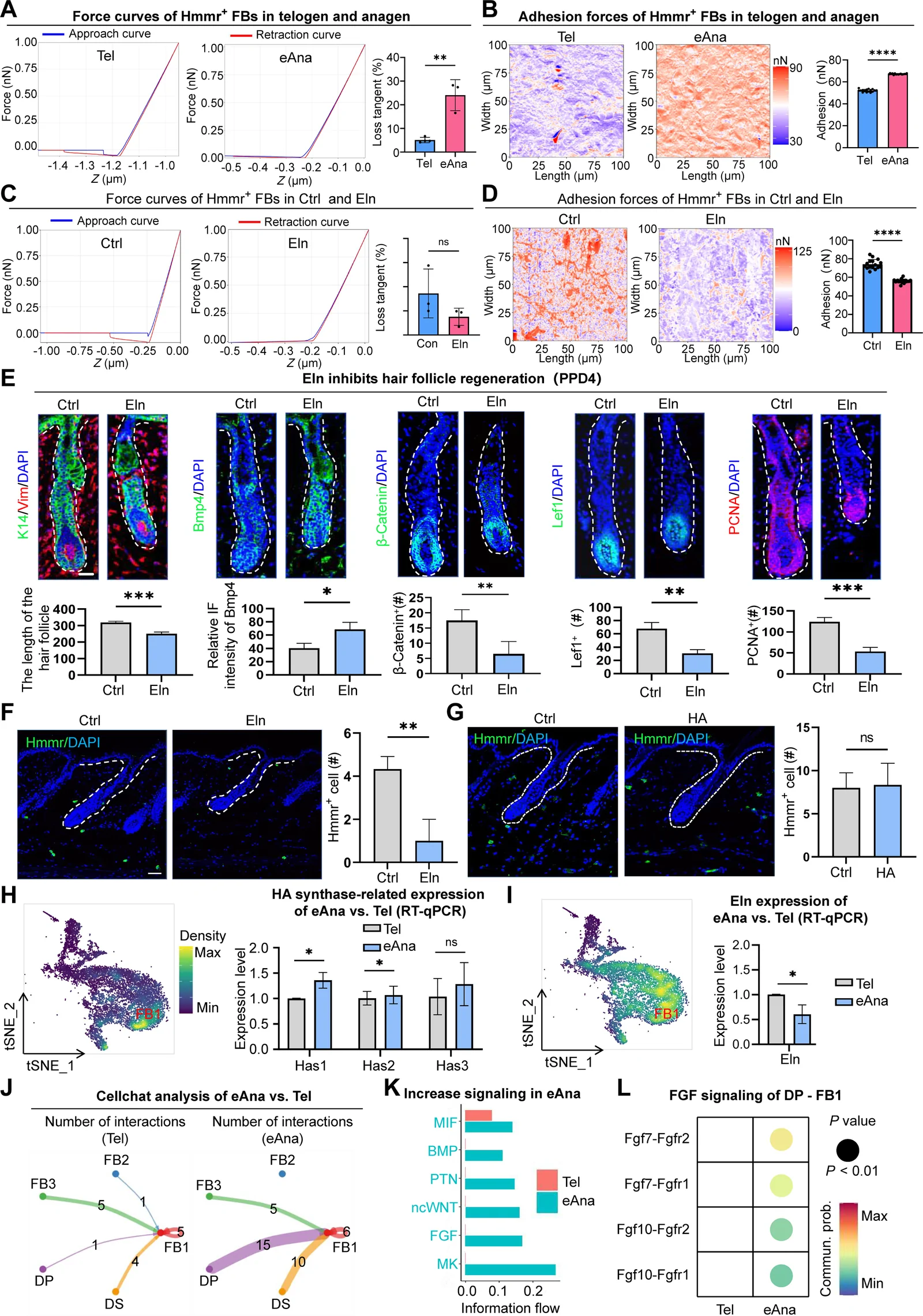
Mechanical crosstalk between fibroblasts and ECM directs follicle regeneration. (A) Representative loading–unloading curves and corresponding loss coefficients of ECM in HMMR^+^ fibroblast regions at telogen and early anagen stages detected by atomic force microscopy (AFM). (B) AFM-based quantitative analysis of ECM adhesion force in HMMR^+^ fibroblast regions at telogen and early anagen stages (*n* = 16; *P* < 0.05, **P < 0.01, ***P < 0.001). (C) Representative loading–unloading curves and corresponding loss coefficients of ECM in HMMR^+^ fibroblast regions following subcutaneous Eln treatment detected by AFM. (D) AFM-based quantitative analysis of ECM adhesion force in HMMR^+^ fibroblast regions following subcutaneous Eln treatment (*n* = 16; *P* < 0.05, **P < 0.01, ***P < 0.001). (E) IF staining demonstrating the effect of subcutaneous Eln injection on the expression of Bmp4, β-catenin, Lef1, and PCNA (PPD4). Scale bar, 25 μm. (F and G) IF staining reveals the effects of subcutaneous Eln and HA injections on Hmmr expression. Scale bar, 50 μm. (H and I) Gene scoring analysis indicates that HA synthesis and Eln expression are predominantly localized in the FB1 cell subpopulation. (J) CellChat analysis compares the interaction numbers between dermal cell subpopulations and FB1 in telogen and early anagen hair follicles. (K) Information flow of interactions between dermal cell subpopulations and FB1 in telogen and early anagen hair follicles. (L) Bubble plot illustrates FGF signaling pathways in DP–FB1 interactions. Mean ± SEM (*n* = 3). Unpaired 2-tailed *t* test. *P < 0.05, **P < 0.01, ***P < 0.001.

Under homeostatic conditions, dermal fibroblasts maintain optimal ECM mechanical properties, including stiffness and viscoelasticity, to support normal tissue function. HA and elastin (Eln), 2 major ECM components critical for biomechanical homeostasis, undergo substantial remodeling during the telogen-to-anagen transition. IF staining analysis revealed strong up-regulation of HA accompanied by a marked decrease in Eln expression (Fig. S4A and B). To further quantify the dynamic viscoelastic changes of the local microenvironment [4], we measured loading–unloading curves and adhesion force of ECM specifically in HMMR^+^ fibroblast regions by atomic force microscopy (AFM). Quantifications of both adhesion force and loss coefficient of ECM in HMMR^+^ fibroblast regions were greatly elevated in the early anagen stage compared with the telogen stage (Fig. 4A and B). Combined with the altered HA and Eln expression patterns, these biomechanical data suggest that the ECM viscoelasticity of HMMR^+^ fibroblast microdomains is markedly increased during early anagen, which closely correlates with the initiation of hair regeneration.

To elucidate the functional distinctions between HA and Eln, we performed GO enrichment analysis on HA synthase-expressing fibroblasts (Has1^+^ and Has2^+^) and compared them with Eln^+^ fibroblasts. Has1^+^ fibroblasts were enriched in terms related to ECM reorganization and collagen synthesis, whereas Has2^+^ fibroblasts were associated with epidermal proliferation and skin development (Fig. S4C and D). In contrast, genes down-regulated in Eln^+^ fibroblasts were linked to hair cycle regulation (Fig. S4E), indicating that HA promotes ECM remodeling and regeneration, while Eln may act to restrain these processes.

To further determine how exogenous HA and Eln modulate ECM biomechanical properties in the HMMR^+^ fibroblast microenvironment during hair regeneration, we again used AFM to measure measured loading–unloading curves and adhesion force, and calculated the corresponding loss coefficient of ECM within HMMR^+^ fibroblast regions following subcutaneous HA or Eln injection. Our analyses demonstrated that HA treatment pronouncedly increased the ECM adhesion force and loss coefficient in HMMR^+^ fibroblast areas, whereas Eln treatment exerted the opposite effects and markedly reduced these viscoelastic parameters (Fig. 4C and D and Fig. S4F and G).

Further validation via bulk RNA-seq and qRT-PCR showed that exogenous Eln injection led to significant down-regulation of pro-regenerative factors (e.g., β-catenin, Lef1, Wnt10a, and Shh) and proliferation markers (Pcna and Mki67), along with up-regulation of the inhibitory factor Bmp4 (Fig. S4H and I). These expression changes were corroborated by IF staining (Fig. 4E and Fig. S4K). Notably, Eln injection also markedly reduced expression of the HA receptor Hmmr, whereas HA injection showed no such effect (Fig. 4F and G and Fig. S4J). These results not only suggest that increased ECM viscoelasticity may facilitate hair regeneration through activation of HMMR signaling but also validate our previously proposed hypothesis that Hmmr functions as a mechanosensor, and further clarify the functional localization of ECM viscoelastic remodeling and Hmmr^+^ FBs in regulating hair regeneration, with the former serving as the effector and the latter as the sensor, which together drive the hair regeneration process.

We next sought to identify upstream regulators that modulate ECM viscoelasticity during regeneration. Intriguingly, bioinformatic analysis identified a fibroblast subpopulation (FB1) that coexpresses HA synthases (Has1/2/3) and Eln (Fig. 4H and I). Moreover, CellChat analysis indicated enhanced interaction between DP and FB1 during regeneration (Fig. 4J and Fig. S4L and M), specifically through activation of the FGF signaling pathway (Fig. 4K and L). Previous studies have implicated FGF signaling in the suppression of elastogenic expression and the organized assembly of elastic ECM in other tissues [39,40]. Based on these findings, we propose a model in which DP-derived FGF signaling suppresses Eln expression in FB1 fibroblasts, thereby increasing ECM viscoelasticity. This mechanical alteration in turn activates HMMR signaling to promote hair regeneration.

### Increased ECM viscoelasticity promotes hair regeneration in mouse skin organoids

**Fig. 5.**
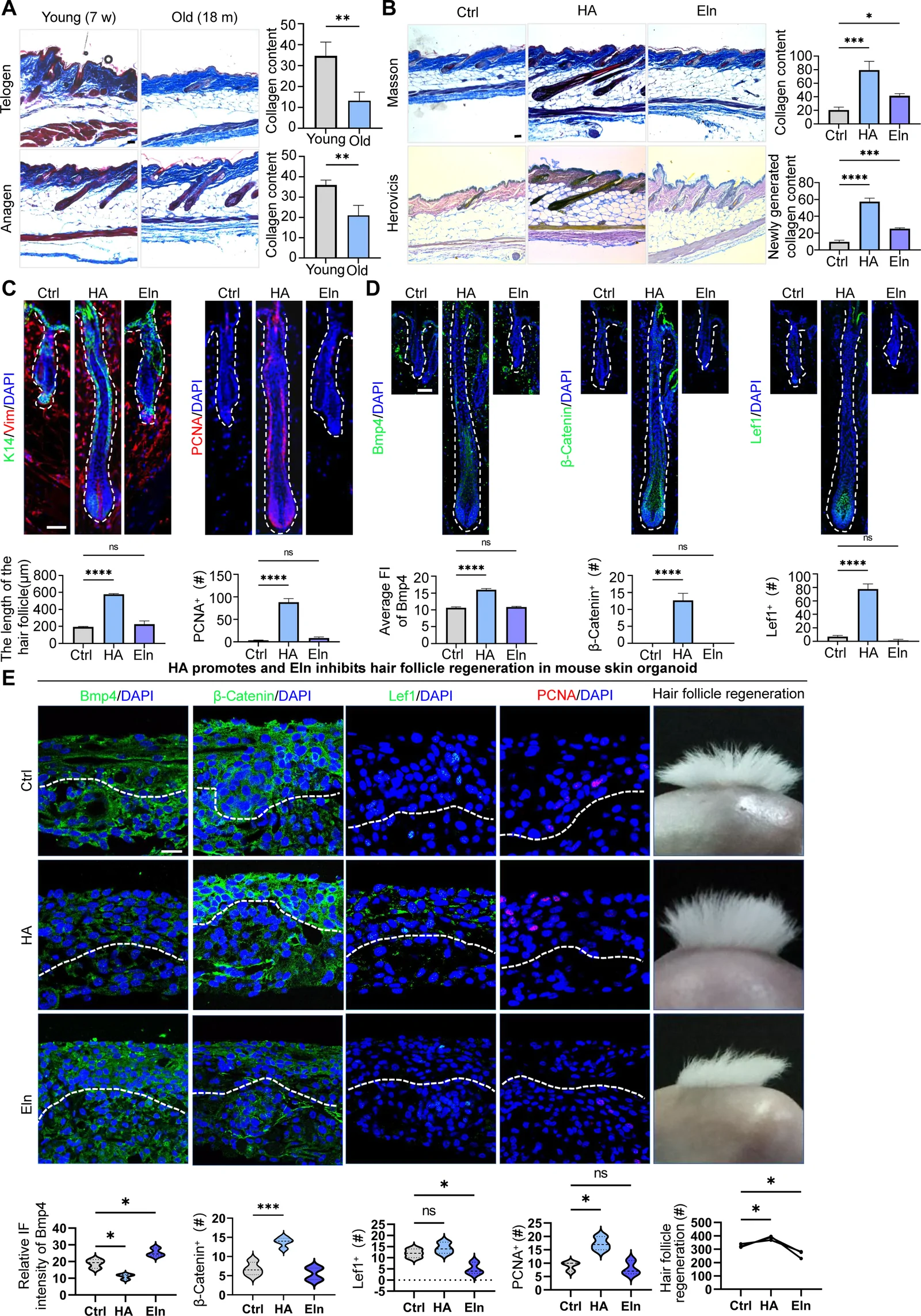
Regulation of hair regeneration by increased ECM viscoelasticity in mouse skin organoids. (A) Masson staining showing collagen distribution in the ECM of hair follicles at telogen and anagen phases in young and old mouse. Scale bar, 50 μm. (B) Masson and Herovici staining showing the effect of subcutaneous injection of HA and Eln on collagen regeneration in the skin. Scale bar, 50 μm. (C and D) IF staining highlighting the impact of subcutaneous injection of HA and Eln on the expression of Bmp4, β-catenin, Lef1, and PCNA. Scale bar, 50 μm. (E) Left: IF staining showing the effects of HA and Eln on the expression of Bmp4, β-catenin, Lef1, and PCNA in the mouse skin organoid model. Right: Quantitative comparison of hair regeneration after transplantation into nude mice between HA/Eln treatment groups and the control group. Scale bar, 25 μm. Mean ± SEM (*n* = 3). Unpaired 2-tailed *t* test or one-way ANOVA with Tukey’s post hoc test. *P < 0.05, **P < 0.01, ***P < 0.001.

Aging is accompanied by loss of ECM structural integrity, including fragmented collagen components and disorganized elastic fiber networks [41,42]. To investigate how ECM viscoelasticity, modulated by HA and Eln, influences hair regeneration in this context, we first examined collagen content across age groups using Masson’s trichrome staining. Compared with young skin, aged follicles displayed a marked reduction in collagen fiber density in both telogen and anagen phases (Fig. 5A). We then modulated dermal viscoelasticity through subcutaneous injection of HA or Eln in mouse models. Masson and Herovici staining revealed that HA treatment not only increased collagen content and the collagen neogenesis index but also promoted anagen entry.

In contrast, Eln enhanced collagen synthesis without triggering regeneration (Fig. 5B), suggesting that hair regeneration depends not merely on collagen abundance, but specifically on viscoelasticity-associated biomechanical cues. Consistently, IF analysis showed that HA treatment suppressed BMP4 while up-regulating β-catenin, LEF1, and PCNA. Eln administration produced the opposite effect, elevating BMP4 and inhibiting Wnt pathway components (Fig. 5C and D). These data support a model in which increased ECM viscoelasticity promotes regeneration by tilting the balance from BMP inhibition toward Wnt activation.

We subsequently confirmed the presence of HA and Eln in mouse skin organoids (Fig. S5A to C). GO enrichment further showed that both Has2^+^ and Eln^+^ fibroblasts are associated with ECM organization pathways (Fig. S5D and E). Notably, HA-treated organoids recapitulated the signaling shifts observed in vivo and exhibited an approximately 30% increase in hair follicle density post-transplantation, whereas Eln-treated organoids showed opposing phenotypes (Fig. 5E and Fig. S5F and G). Together, these results establish that increased ECM viscoelasticity enhances hair regeneration across both in vivo and organoid models, acting through a BMP/Wnt signaling axis.

### Hmmr^+^ FBs interact with DP by the Ncam1–Fgfr1 pathway to promote hair regeneration

**Fig. 6.**
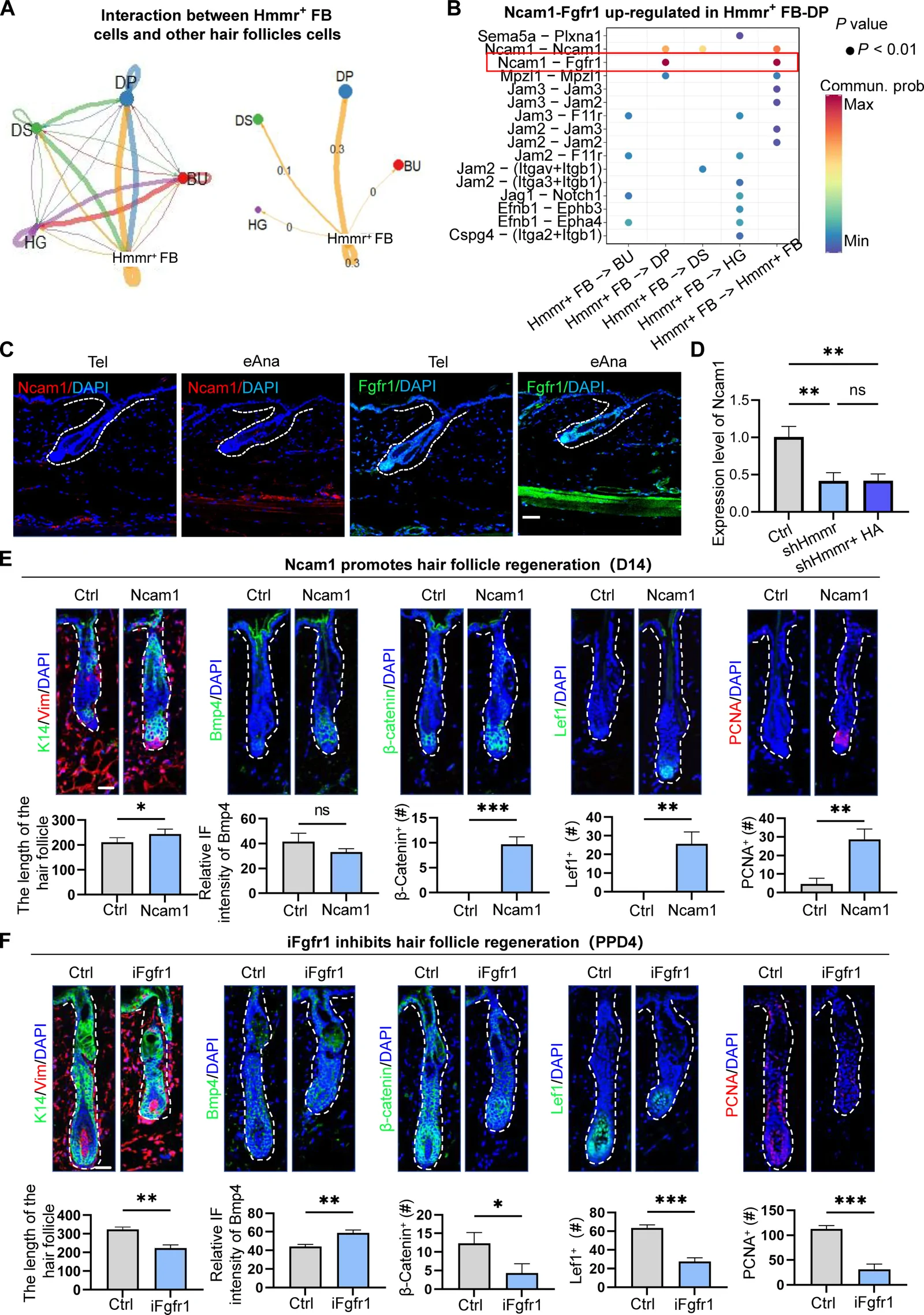
Hmmr^+^ FBs interact with DP by the Ncam1–Fgfr1 pathway to promote hair regeneration in vivo. (A) Schematic illustrating the interaction network of Hmmr^+^ FBs with hair regeneration-related cell subpopulations during the early anagen phase. (B) Bubble plot displaying signaling pathways associated with Hmmr^+^ FBs–DP interactions. (C) IF results showing differential expression of Ncam1 and Fgfr1 between telogen and early anagen hair follicle. Scale bar, 50 μm. (D) qRT-PCR showing the relative expression level of Ncam1 after Hmmr knockdown. (E) IF staining demonstrating the effects of subcutaneous Ncam1 injection on Bmp4, β-catenin, Lef1, and PCNA expression (day 14). Scale bar, 25 μm. (F) IF staining showing the effects of subcutaneous injection of Fgfr1 inhibitor PD173074 on Bmp4, β-catenin, Lef1, and PCNA expression (PPD4). Scale bar, 25 μm. Mean ± SEM (*n* = 3). Unpaired 2-tailed *t* test or one-way ANOVA with Tukey’s post hoc test. *P < 0.05, **P < 0.01, ***P < 0.001.

To delineate the molecular mechanisms by which Hmmr regulates hair regeneration, we performed CellChat analysis to map intercellular communication networks. This revealed that Hmmr^+^ FBs exhibits the strongest interaction with DP during early anagen (Fig. 6A), primarily mediated by the NCAM1–FGFR1 signaling axis (Fig. 6B). Consistent with this, qRT-PCR showed coordinated up-regulation of Ncam1 and Fgfr1 during regeneration (Fig. S6A), and IF confirmed that NCAM1 is enriched in Hmmr^+^ FBs, whereas FGFR1 localizes to DP cells (Fig. 6C). Critically, both shRNA-mediated Hmmr knockdown and pharmacological inhibition of HMMR with 4-MU or triptolide markedly down-regulated Ncam1 and Fgfr1 expression, as validated by qRT-PCR and IF staining (Fig. 6D and Fig. S6B and C). These results indicate that HMMR activity is required for maintaining the NCAM1–FGFR1 signaling crosstalk between Hmmr^+^ FBs and DP cells during hair regeneration.

Functional enrichment analysis further supported these findings: Ncam1 was associated with “Cell cycle” regulation (Fig. S6D), and Fgfr1 was linked to “hair follicle development” and “epidermis development” (Fig. S6E). To functionally validate this axis in vivo, we exogenously supplemented recombinant NCAM1 protein and PD173074, an inhibitor of FGFR1, respectively. NCAM1 protein treatment markedly down-regulated the expression of BMP4 and up-regulated the levels of β-catenin, LEF1, and PCNA, indicating that NCAM1 facilitates hair follicle regeneration (Fig. S6F). In contrast, FGFR1 inhibition by PD173074 suppressed hair regeneration (Fig. S6G). These effects were further validated by IF staining (Fig. 6F and G and Fig. S6H). Collectively, our results demonstrate that the NCAM1–FGFR1 axis modulates BMP and Wnt/β-catenin signaling cascades to govern the hair follicle regeneration process.

**Fig. 7.**
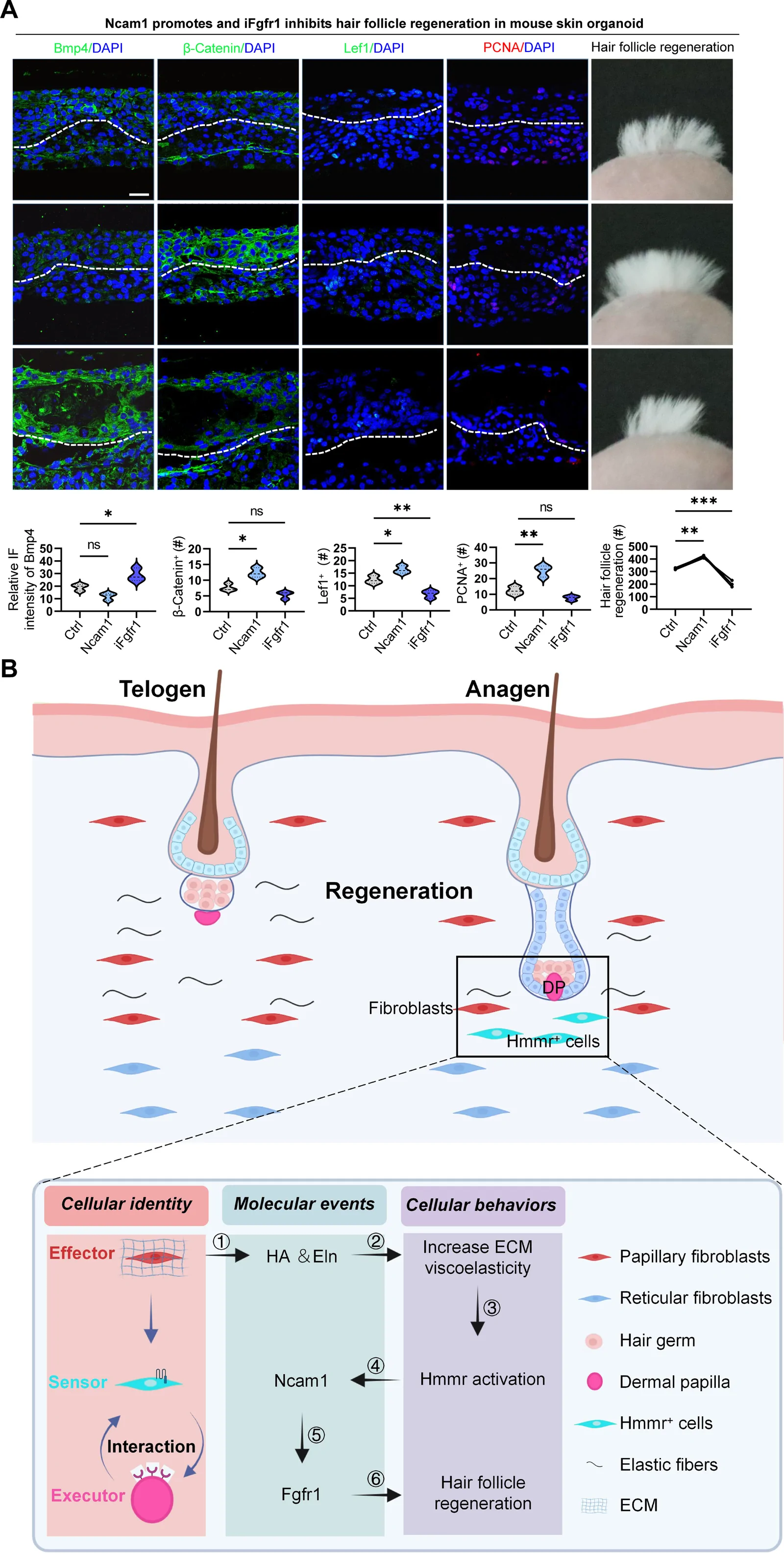
Hmmr^+^ FBs interact with DP via the Ncam1–Fgfr1 pathway to promote hair regeneration in skin organoid models. (A) Left: IF staining shows the effects of Ncam1 and Fgfr1 inhibitor treatments on the expression of Bmp4, β-catenin, Lef1, and PCNA in mouse skin organoid models. Right: Quantification of hair regeneration in nude mice after transplantation following inhibitor treatment. Scale bar, 25 μm. (B) Schematic illustrating the mechanism by which Hmmr promotes hair follicle regeneration: During hair follicle regeneration, the upper dermis (effector) increases the viscoelasticity of the ECM by regulating HA synthesis and Eln expression. This change is sensed by Hmmr^+^ cells (sensor), leading to Hmmr activation. Subsequently, via the Ncam1–Fgfr1 signaling pathway, these cells interact with the DP cells (executor), ultimately promoting hair follicle regeneration. Mean ± SEM (*n* = 3). One-way ANOVA with Tukey’s post hoc test. *P < 0.05, **P < 0.01, ***P < 0.001.

These mechanisms could be recapitulated in skin organoids as well. Spatial transcriptomics and IF confirmed expression of Ncam1 and Fgfr1 across dermal subpopulations (Fig. S7A and B), and enrichment analysis associated them with ECM composition and collagen synthesis (Fig. S7C and D). Functionally, NCAM1-enhanced organoids showed reduced BMP4 levels, elevated β-catenin, LEF1, and PCNA, and generated considerably more hair follicles after transplantation compared to FGFR1-inhibited organoids (Fig. 7A and Fig. S7E and F). Together, these results establish the NCAM1–FGFR1 signaling axis as a key downstream effector of HMMR. This pathway operates through a dual mechanism: It facilitates regeneration by remodeling the pericellular ECM and directly promotes epithelial proliferation by activating the Wnt/β-catenin pathway.

## Discussion

Tissue regeneration is not merely linearly driven by genetic programs, but rather represents an adaptive process dynamically encoded by the biomechanical properties of the microenvironment, decoded through cellular perception, and reshaped via signal transduction [43,44]. For instance, PIEZO1-mediated calcium signaling senses mechanical forces through E-cadherin to reinforce the mechanical properties of hair follicle stem cells (HFSCs) and maintain their quiescence [9], while microRNA-205 fine-tunes actomyosin contractility and cell mechanics by targeting cytoskeletal and adhesion genes to promote hair regeneration [8]. In this study, we focus on the dynamic viscoelastic remodeling of the ECM during the telogen-to-anagen transition and identifies a novel tripartite biomechanical regulatory module consisting of ECM mechanical effectors, Hmmr^+^ fibroblast mechanical sensors, and DP signal executors, which collaboratively drives hair follicle regeneration. In particular, we verified the dynamic biomechanical alterations of the peri-follicular microenvironment via AFM. AFM quantification of loading–unloading curves, loss coefficients, and independent adhesion force confirmed that the ECM viscoelasticity and adhesion properties in Hmmr^+^ FB regions are greatly elevated in early anagen compared to telogen. This mechanical remodeling is attributed to the reciprocal expression changes of core ECM components: Hair follicle regeneration initiation is accompanied by up-regulation of HA and down-regulation of Eln. Subsequent experiments with exogenous pharmacological and genetic manipulations further validated the independent regulatory functions of HA and Eln, showing that subcutaneous HA treatment increased ECM loss coefficients and adhesion force to enhance microenvironmental viscoelasticity, whereas Eln supplementation exerted opposite biomechanical effects and reduced ECM viscoelasticity. These mechanical alterations further determine the activation state of downstream regenerative signaling. Eventually, we identified the Hmmr^+^ FBs as a primary mechanical sensor that bridges the ECM-derived mechanical information, the effector, from the peri-follicular microenvironment and biochemical information in the DP via a reciprocal HA-HMMR and NCAM1–FGFR1 signaling crosstalk, thereby coordinately driving the telogen-to-anagen transition of hair follicles.

Although dermal fibroblasts are long known as the primary producers of the ECM [5,45], their functional heterogeneity and spatiotemporal regulation of their surrounding mechanical microenvironment during tissue regeneration remain incompletely understood. Through integrated single-cell transcriptomic analysis, we identified a unique fibroblast subpopulation, which we termed Hmmr^+^ FBs, that emerges specifically during early follicle regeneration. Hmmr^+^ FBs are characterized by high expression of HMMR and exhibit transcriptional similarity to DP, suggesting a potential DP-related lineage origin and a role as a sensory interface linking the DP with the surrounding mechanical microenvironment. Similarly, research has shown that upon forced β-catenin activation, hair follicle dermal stem cells within the DS can be recruited to repopulate both the DS and DP [46]. This finding critically underscores the remarkable plasticity of dermal fibroblasts, demonstrating that non-DP fibroblasts can be reprogrammed to acquire the identity and function of DP cells. Our findings further expand the spectrum of dermal fibroblast plasticity, demonstrating that Hmmr^+^ FBs act as indispensable mechanical sensors for hair cycle transition. Functionally, we verified that Hmmr^+^ FBs rely on the HA-HMMR axis to perceive ECM viscoelasticity changes and initiate downstream intercellular communication. Hmmr knockdown in skin tissues markedly down-regulated the expression of Ncam1 in Hmmr^+^ FBs, confirming that HMMR is an essential upstream regulator of NCAM1 signaling. These results solidify the mechanotransduction cascade from ECM mechanical alteration to cell fate determination.

Notably, HMMR is not just a canonical adhesion receptor but rather a mechanosensitive motility receptor whose expression correlates with a highly proliferative state. Indeed, we found that increased ECM viscoelasticity, triggered by Eln down-regulation, specifically up-regulates HMMR in Hmmr^+^ FBs, indicating that HMMR serves as both a sensor and a biomechanical responder to ECM mechanical cues. This mechanism resembles the HA-HMMR-FAK/Src-mediated epithelial–mesenchymal transition (EMT) program during zebrafish epicardial regeneration [47], suggesting that the HA-HMMR axis may represent a universal mechano-regenerative module operating across species and organs.

Traditionally, the ECM has been viewed as a structural scaffold, with its mechanical functions often simplified to stiffness or elastic modulus [48], while viscoelasticity has long been neglected in tissue regeneration research. However, growing evidence has suggested that ECM viscoelasticity, specifically its time-dependent stress relaxation and energy dissipation capacity, is the key physical parameter governing how cells sense the mechanical microenvironment and make fate decisions [3,49]. Our study further supports this observation by demonstrating that during the telogen-to-anagen transition, up-regulation of HA synthesis coupled with down-regulation of Eln expression pronouncedly increases ECM viscoelasticity, indicating that ECM softening is a prerequisite for regeneration initiation. Mechanistically, HA confers rapid stress-relaxation capacity to the ECM via its highly hydrated polysaccharide network crosslinked with proteoglycans, whereas Eln enhances elastic recoil through lysyl oxidase-mediated crosslinking, forming elastic fibers [39]. The dynamic balance between these 2 components acts as a “molecular switch” controlling ECM viscoelasticity. This indicates that the follicular microenvironment not only passively provides mechanical support but also actively sculpts its biomechanical properties to guide the regenerative program.

Previous studies have established that the mechanical properties of the ECM regulate tissue homeostasis through mechanotransduction, and that the spatial communication network established via ligand-receptor interactions between the DP and surrounding cells is critical for follicular development [50,51]. Building on this foundation, this study focused on the specific mechanism by which mechanical signals are converted into chemical signals during hair regeneration. We discovered that when ECM viscoelasticity increases, the Hmmr^+^ FBs subpopulation located in the sub-DP region perceives this physical signal change through the mechanosensitive receptor HMMR, leading to the up-regulation of NCAM1 expression, thereby achieving the primary conversion from mechanical to chemical signaling. This conversion relies on the specific binding of NCAM1 to FGFR1 on the surface of DP cells. This signaling conversion process is accompanied by enhanced cell proliferation activity and up-regulation of regeneration-associated genes such as β-catenin and Lef1, forming a complete mechano-chemical signal transduction cascade. These findings demonstrate that the mechanical properties of the ECM are translated into biochemical instructions guiding cyclical hair follicle remodeling, which is particularly important during anagen. During this phase, the highly viscoelastic ECM during hair regeneration poses a physical barrier that impedes the downward growth of the hair follicle. Based on our data, we believe that the DP located at the base of the follicle dynamically modulates ECM composition by secreting specific signaling molecules, thereby reducing elastic recoil. This spatiotemporal remodeling of the ECM is precisely sensed by Hmmr^+^ FBs positioned in this region via their highly expressed HMMR receptor. As a mechanosensitive motility receptor, HMMR not only detects the increase in ECM viscoelasticity but also transduces this mechanical information into pro-proliferative biochemical instructions through activation of the downstream NCAM1–FGFR1 pathway. This mechanism spatially guides the downward progression of the hair follicle and temporally ensures synchronized regeneration initiation with softening of its surrounding microenvironment.

Our study has several limitations. While our AFM measurements capture dynamic changes in adhesion and mechanical energy dissipation within HMMR^+^ fibroblast microdomains, they do not provide a complete mechanical characterization of the ECM. In addition, although functional inhibition and shRNA-mediated knockdown experiments strongly suggest a signaling cascade initiated by HMMR-dependent sensing of HA followed by NCAM1–FGFR1 pathway, it does not fully distinguish the mechanical and biochemical contributions of the HA–HMMR interaction. Given that HA can simultaneously alter ECM physical properties and engage HMMR as a ligand, whether these 2 modes of regulation act cooperatively or via a linear pathway requires further investigation.

In summary, this study identifies a previously unrecognized fibroblast subpopulation, Hmmr^+^ FBs, as a key sensor and transmitter of biomechanical signals during hair regeneration. This signaling module is comprised of ECM (effector), Hmmr^+^ FBs (sensor), and DP (executor), where Hmmr^+^ FBs sense changes in ECM viscoelasticity through HMMR and transmit these cues to DP cells via NCAM1–FGFR1 signaling, thereby activating and maintaining regenerative programs. Our results underscore the centrality of mechanical cues in driving dynamic regeneration, cellular perception, and signal decoding within the niche, providing a paradigm for studying other tissue regeneration.

## Materials and Methods

### Ethics statement

All experimental procedures were approved by the Institutional Animal Care and Use Committee of Chongqing University (approval no. CQCH-LAE-A0000202055). Animal husbandry and experimentation were conducted in strict accordance with the Guidelines for the Care and Use of Laboratory Animals and institutional animal-welfare requirements.

### Mouse shaving model

Seven-week-old female C57BL/6J mice were selected and randomly divided into groups, with 3 mice in each group. Each group was treated with either physiological saline or a recombinant protein/large molecule. Each mouse, weighing approximately 20 g, was administered a 140-μl intraperitoneal injection of a 1% sodium pentobarbital solution. Using the limbs as boundaries, the hair on the mouse’s back was removed using a depilatory cream. The appearance of entirely pink back skin indicated that the hair follicles were in the telogen phase, thus meeting the experimental requirements.

### Mouse plucking model

Seven-week-old female C57BL/6J mice were selected and randomly divided into experimental groups, with at least 3 mice per group. Each group was treated with either physiological saline or an inhibitor. Each mouse, weighing approximately 20 g, was administered a 140-μl intraperitoneal injection of a 1% sodium pentobarbital solution. Using the limbs as boundaries, molten paraffin wax was applied evenly from the head to the tail on the mouse’s back using a Pasteur pipette. The temperature of the wax was ensured to be appropriate to avoid burning the mouse’s skin. Once the wax had solidified, the waxed area was sequentially removed from tail to head to synchronize the hair follicles into the growth phase. This synchronization was essential for follicle inhibition experiments.

### Newborn mouse skin organoid culture

Within 24 h of birth, the dorsal skin of newborn mice was immersed in a 0.25% trypsin solution at 4 °C overnight to separate the dermis from the epidermis. The epidermal cells were obtained through cutting with scissors, followed by filtering and centrifugation. The dermal cells were digested with 0.35% collagenase I for 15 to 20 min, then filtered and centrifuged. The separated epidermal and dermal cells were mixed in specific proportions and carefully deposited into the upper chamber of a Transwell culture system. The lower chamber was filled with 750 μl of Dulbecco’s modified Eagle’s medium (DMEM) culture medium containing 10% fetal bovine serum. The cells were cultured in a 5% CO₂ atmosphere at 37 °C, and the culture medium was refreshed every other day.

### Newborn mouse skin organoid transplantation

We defined fully competent day 4 (D4) skin organoids as the graft material for implantation; these organoids reach the coalescence morphogenetic stage with a 65.4% coalescence rate, accompanied by basal YAP1 activation, elevated Wnt10a/MMP expression, and basement membrane remodeling, which endows them with hair follicle regeneration capacity post-transplantation. Only organoids satisfying this unified morphological maturity standard were utilized. Male Balb/C nude mice that are 7 weeks old or older, with a weight of approximately 20 to 25 g, are selected. Each nude mouse, weighing 20 g, is administered an intraperitoneal injection of 140 μl of 1% sodium pentobarbital solution and 25 μl of 25% flunixin meglumine for pain relief. After the nude mouse is completely anesthetized, its back is disinfected with iodophor solution, repeating the process 3 times. The skin organoids from a 12-well plate are removed and placed cell side up in the culture medium for later use. A wound that matches the size of the organoid is cut on the side of the nude mouse’s back. One standardized mature D4 organoid was implanted per individual animal, and all organoids utilized within a single experimental cohort were sourced from an identical culture batch to eliminate batch-to-batch variability across experimental groups. Once the cell side of the organoid is attached to the wound, the organoid is sutured to the skin. Baiduobang ointment is then evenly applied to the wound to inhibit infection. The back and abdomen are wrapped with a bandage for fixation. Finally, the nude mouse’s skin, adhesive bandage, and bandage are sutured together. Each nude mouse is sutured at 4 positions on both the upper and lower parts of the back and abdomen.

### Small-molecule perturbation

For shaved mice, recombinant proteins or large molecules were subcutaneously injected from day 0 to day 5 post-shaving. Sampling was performed when the follicles were in the early growth phase following treatment. For plucked mice, inhibitor drugs were subcutaneously injected from day 0 to day 3 or day 5 post-plucking, with sampling conducted on day 4 or day 6. The following reagents and concentrations were used: 50 μl of HA solution (2 mg/ml, CAS# HY-B0633A, MCE), 50 μl of Ncam1 solution (1 μg/ml, CAS# HY-P78764, MCE), 50 μl of 4-MU solution (5 mM, CAS# HY-N0187, MCE), 50 μl of triptolide (10 μM, CAS# M3985, Abmole), 50 μl of Eln solution (1 μg/ml, CAS# P1577, FineTest), and 50 μl of PD173074 solution (100 μM, CAS# HY-10321, MCE).

### Hmmr knockdown in mice via subcutaneous injection

Recombinant adenovirus carrying shRNA targeting Hmmr (Ad-sh-Hmmr) and negative control adenovirus (Ad-sh-NC) were constructed and purified. After viral titer determination, the adenoviruses were diluted to the working concentration with sterile phosphate-buffered saline (PBS). Seven-week-old female C57BL/6 mice whose hair follicles were in the telogen phase were randomly assigned to the Ad-sh-Hmmr experimental group and the Ad-sh-NC control group. Following dorsal hair depilation, 50 μl of adenovirus suspension at a dosage of 1 × 10^9^ plaque-forming units (PFU) per mouse was subcutaneously and evenly injected into the depilated dorsal region of each mouse, while mice in the control group received an equal volume of Ad-sh-NC solution. Dorsal skin tissues were harvested at predetermined time points for subsequent IF staining, qRT-PCR, and histological analyses.

### Hmmr knockdown in cultured mouse skin organoids

When the organoids had been cultured for 6 to 12 h, the medium was replaced with fresh induction medium supplemented with Ad-sh-Hmmr or Ad-sh-NC for adenoviral transduction at a multiplicity of infection (MOI) of 10. After 12 h of incubation with the adenoviruses, the virus-containing medium was discarded. The organoids were washed twice with sterile PBS and then cultured continuously in standard complete organoid medium.

### Tissue and cell sampling

#### Mouse back skin sampling

The mouse skin was cut along the direction of the hair follicles, flattened on filter paper, and fixed in 4% paraformaldehyde (PFA) for 48 h.

#### Skin organoid sampling

The 12-well culture plate was removed, the medium was aspirated from the wells, and the wells were washed with PBS. An appropriate amount of 4% PFA was added to the lower chamber of each well. The plate was placed in a refrigerator at 4 °C for 12 h. Subsequently, an appropriate amount of 4% PFA was slowly added to the upper chamber, covering the cells. Fixation was continued in the refrigerator at 4 °C for an additional 4 h.

### AFM mechanical characterization

All dermal ECM mechanical measurements of hair follicle tissues were performed using an Asylum Research MFP-3D Origin AFM system (Oxford Instruments) equipped with closed-loop scanners, GetReal auto-calibration module, and liquid cell chamber. Hair follicle tissues were embedded in optimal cutting temperature compound (OCT) compound, cryosectioned to a uniform thickness of 30 μm, and immersed in precooled PBS buffer at room temperature (25 °C) throughout all AFM measurements to maintain native tissue hydration and physiological microenvironment.

#### Force volume mapping for loading–unloading curves, adhesion force, and hysteresis quantification

Force volume mode was applied to record full indentation loading–unloading force–distance curves for quantitative viscoelastic and adhesion analysis. Rectangular silicon cantilevers (Olympus BL-AC40TS) with a nominal tip radius of 8 nm were utilized. Cantilever spring constants were calibrated via the thermal noise method prior to all experiments to ensure force measurement accuracy (nominal *k* = 0.1 N/m). Scanning area was set to 100 × 100 μm^2^ with a pixel resolution of 256 × 256, corresponding to a spatial sampling of ~0.39 μm per pixel. Scan frequency was fixed at 0.5 Hz; tip approach and retraction speeds were both maintained at 3 μm/s. The maximum indentation loading force was limited to 2.5 nN, with a 50-ms dwell contact time once the target force threshold was reached. Raw force–distance curves containing full loading and retraction trajectories were exported and processed using Nanoscope Analysis software. The minimum force value on each retraction curve was extracted to calculate ECM adhesion force magnitude. Hysteresis area enclosed by paired loading–unloading curves was quantified as the readout of viscous energy dissipation, a core metric reflecting ECM viscoelasticity.

#### Quantitative nanomechanical mapping

Complementary nanomechanical imaging was conducted in quantitative nanomechanical mapping (QNM) mode using Bruker RTESP-300 cantilevers (nominal spring constant = 40 Nm^−1^, resonance frequency = 300 kHz). Scanning size was 100 × 100 μm^2^ at 256 × 256-pixel resolution with a scan rate of 1 Hz, and peak-to-peak oscillation amplitude was set to 0.5 V. The setpoint ratio was fixed at 0.7 to hold cantilever oscillation at 70% of its free amplitude; integral feedback gain = 0.2 and proportional feedback gain = 0.5 were applied for stable tip-sample tracking. Surface topography, elastic modulus distribution, and adhesion force maps were acquired synchronously during scanning. Mechanical modulus values were derived by fitting indentation curves to the Derjaguin–Müller–Toporov (DMT) contact mechanics model, which accounts for both elastic deformation and surface adhesion forces at the nanoscale tissue interface.

### H&E staining

For 8-μm paraffin sections, deparaffinization was performed twice with xylene for 15 min each, followed by hydration using a graded series of alcohol (100%, 100%, 95%, 85%, 75%, and 50%). The sections were then immersed in double-distilled water for 3 min. Staining was conducted with hematoxylin solution for 2 min, followed by rinsing with tap water. The sections were differentiated in a differentiation solution for 10 s and rinsed again in tap water until they turned blue. Counterstaining was performed with eosin for 2 min and 30 s, followed by rinsing with tap water. Dehydration was carried out quickly using a graded series of alcohol, and clearing was done in xylene for 1 min. Finally, the sections were mounted with neutral resin, and images were captured using a microscope.

### Masson staining

After deparaffinization and hydration of the 8-μm paraffin sections, the following steps were performed in sequence: Weigert’s iron hematoxylin staining solution was applied for 7 min, followed by differentiation with an acidic differentiation solution for 10 s. The sections were then blued with Masson’s bluing solution for 4 min, stained with fuchsin staining solution for 7 min, washed with a weak acid working solution for 30 s, and treated with phosphomolybdic acid solution for 1 min and 30 s. The sections were washed again with the weak acid working solution for 30 s, stained with aniline blue staining solution for 1 min and 30 s, and washed with the weak acid working solution for another 30 s. The sections were then quickly dehydrated with graded alcohols, immersed in xylene for 1 min, and finally mounted with neutral resin. Images were captured using a microscope.

### Immunofluorescence

The 8-μm paraffin sections were deparaffinized and hydrated. The samples were immersed in an antigen retrieval solution composed of citric acid and sodium citrate. Separation of the samples was performed using a histological pen, followed by blocking with 2% bovine serum albumin at 37 °C for 1 h to reduce nonspecific binding. The samples were incubated overnight at 4 °C with the primary antibody. Subsequently, the samples were incubated with the corresponding fluorescent secondary antibody at 37 °C for 2 h, followed by incubation with diamidino-2-phenylindole (DAPI) at room temperature for 30 min. Finally, an antifade mounting agent was added and the slides were sealed.

### Image acquisition and fluorescence intensity quantification

All IF images were acquired using a confocal laser scanning microscope. For each antibody used in this study, identical laser power, gain, and offset settings were maintained across all experimental groups to ensure comparability of fluorescence signals. For each biological replicate, at least 3 randomly selected fields of view within the regions of interest (ROIs) were captured under identical acquisition parameters, with field selection performed blinded to the experimental groups. Image analysis and fluorescence intensity quantification were performed using ImageJ software. Briefly, ROIs were manually delineated around the target regions based on the corresponding marker staining, and the mean fluorescence intensity (MFI) of the target protein was measured within the defined ROIs. Background fluorescence was subtracted using an unstained or isotype-matched control section processed in parallel. MFI values for the target proteins were then normalized to the DAPI signal area to correct for variations in tissue section thickness and mounting. All quantification procedures were applied uniformly across all experimental groups for each antibody, and all representative images shown in the figures were derived from the same experimental batches as the corresponding quantified data.

### scRNA-seq data analysis

scRNA-seq data from 3- and 4-week-old dorsal skin hair follicles were obtained from the Gene Expression Omnibus (GSE) dataset. Data quality control (QC), normalization, and feature selection were performed using Seurat v.4.1.1 software. Dimensionality reduction was achieved using t-distributed stochastic neighbor embedding (t-SNE) to cluster cells based on expression patterns and to discover potential cell types. The identities of each cell subgroup were annotated using the CellMarker database and knowledge from the field of skin hair follicle research to determine their cell types and functional features. Intercellular interaction analysis was conducted using CellChat to explore communication networks among different cell populations. scRNA-seq data of neonatal mouse skin organoids originated from prior work in our laboratory (GSE215980). Analysis of dimensionality reduction and clustering was performed as described above. GO and KEGG pathway enrichment analysis was primarily conducted through the online platform https://www.bioinformatics.com.cn for data analysis and visualization.

### Transcriptome analysis

Skin samples were collected from the control group, as well as from groups treated with the 4-MU inhibitor and Eln protein, 4 days after plucking. High-throughput transcriptome sequencing was performed on these skin samples. Library construction and sequencing were conducted at LingEn Biotechnology Co. Ltd., following the manufacturer’s instructions. Extensive RNA-seq data analysis was carried out on the Majorbio Cloud Platform (https://www.majorbio.com).

### Statistical analyses

All quantitative data are presented as mean ± standard error of the mean (SEM). The statistical unit is defined as the independent biological replicate (*n*), which represents an individual animal for in vivo experiments or an individual organoid for organoid culture experiments, unless otherwise specified in the figure legends. For each biological replicate, multiple technical replicates were measured and averaged to generate a single value for that replicate before statistical analysis, thereby avoiding pseudoreplication. All experiments were performed with at least 3 independent biological replicates per group.

Statistical analyses were performed using SPSS software. Comparisons between 2 groups were assessed using unpaired 2-tailed Student’s *t* test. For comparisons involving 3 or more groups, one-way analysis of variance (ANOVA) was performed, followed by Tukey’s honestly significant difference (HSD) post hoc test for multiple comparisons. Prior to parametric testing, data were assessed for normality using the Shapiro–Wilk test and for homogeneity of variances using Levene’s test; data that met these assumptions were analyzed as described above. All image quantification and field selection were performed blinded to the experimental groups to minimize observer bias. *P* value < 0.05 was considered statistically significant (*P < 0.05, **P < 0.01, ***P < 0.001).

## Data Availability

The single-cell RNA-sequencing data analyzed in this study were generated in an accompanying study by our group, which is currently under review. The data have been deposited in the NCBI Gene Expression Omnibus (GEO) under accession number GSE333781 and will be made publicly available upon publication of the accompanying study.

## References

[1] Yanagisawa H, Yokoyama U. Extracellular matrix-mediated remodeling and mechanotransduction in large vessels during development and disease. Cell Signal. 2021;86: Article 110104. 34339854 10.1016/j.cellsig.2021.110104

[2] Han YL, Wang S, Zhang X, Li Y, Huang G, Qi H, Pingguan-Murphy B, Li Y, Lu TJ, Xu F. Engineering physical microenvironment for stem cell based regenerative medicine. Drug Discov Today. 2014;19(6):763–773. 24508818 10.1016/j.drudis.2014.01.015

[3] Chaudhuri O, Cooper-White J, Janmey PA, Mooney DJ, Shenoy VB. Effects of extracellular matrix viscoelasticity on cellular behaviour. Nature. 2020;584(7822):535–546. 32848221 10.1038/s41586-020-2612-2 PMC7676152

[4] Fan W, Adebowale K, Váncza L, Li Y, Rabbi MF, Kunimoto K, Chen D, Mozes G, Chiu DK, Li Y, et al. Matrix viscoelasticity promotes liver cancer progression in the pre-cirrhotic liver. Nature. 2024;626(7999):635–642. 38297127 10.1038/s41586-023-06991-9 PMC10866704

[5] DeLeon-Pennell KY, Barker TH, Lindsey ML. Fibroblasts: The arbiters of extracellular matrix remodeling. Matrix Biol. 2020;91-92:1–7. 32504772 10.1016/j.matbio.2020.05.006 PMC7434687

[6] Lin X, Zhu L, He J. Morphogenesis, growth cycle and molecular regulation of hair follicles. Front Cell Dev Biol. 2022;10: Article 899095. 35646909 10.3389/fcell.2022.899095 PMC9133560

[7] Zhang B, Chen T. Local and systemic mechanisms that control the hair follicle stem cell niche. Nat Rev Mol Cell Biol. 2024;25(2):87–100. 37903969 10.1038/s41580-023-00662-3

[8] Wang J, Fu Y, Huang W, Biswas R, Banerjee A, Broussard JA, Zhao Z, Wang D, Bjerke G, Raghavan S, et al. MicroRNA-205 promotes hair regeneration by modulating mechanical properties of hair follicle stem cells. Proc Natl Acad Sci USA. 2023;120(22): Article e2220635120. 37216502 10.1073/pnas.2220635120 PMC10235966

[9] Wang J, Fu C, Chang S, Stephens C, Li H, Wang D, Fu YC, Green KJ, Yan J, Yi R. PIEZO1-mediated calcium signaling reinforces mechanical properties of hair follicle stem cells to promote quiescence. Sci Adv. 2025;11(22):eadt2771. 40435254 10.1126/sciadv.adt2771 PMC12118625

[10] Bonnans C, Chou J, Werb Z. Remodelling the extracellular matrix in development and disease. Nat Rev Mol Cell Biol. 2014;15(12):786–801. 25415508 10.1038/nrm3904 PMC4316204

[11] Abreu CM, Marques AP. Recreation of a hair follicle regenerative microenvironment: Successes and pitfalls. Bioeng Transl Med. 2022;7(1): Article e10235. 35079623 10.1002/btm2.10235 PMC8780054

[12] Chermnykh E, Kalabusheva E, Vorotelyak E. Extracellular matrix as a regulator of epidermal stem cell fate. Int J Mol Sci. 2018;19(4):1003. 29584689 10.3390/ijms19041003 PMC5979429

[13] Lei M, Harn HI, Li Q, Jiang J, Wu W, Zhou W, Jiang TX, Wang M, Zhang J, Lai YC, et al. The mechano-chemical circuit drives skin organoid self-organization. Proc Natl Acad Sci USA. 2023;120(36): Article e2221982120. 37643215 10.1073/pnas.2221982120 PMC10483620

[14] Chen P, Cescon M, Bonaldo P. Lack of collagen VI promotes wound-induced hair growth. J Invest Dermatol. 2015;135(10):2358–2367. 25989472 10.1038/jid.2015.187

[15] Chu SY, Chou CH, Huang HD, Yen MH, Hong HC, Chao PH, Wang YH, Chen PY, Nian SX, Chen YR, et al. Mechanical stretch induces hair regeneration through the alternative activation of macrophages. Nat Commun. 2019;10(1):1524. 30944305 10.1038/s41467-019-09402-8 PMC6447615

[16] Zhou A, Zhang Y, Chen W, Ren J, Ning X. Bioorthogonal click-engineered microneedle patch enables adeno-associated virus-mediated dermal acidic fibroblast growth factor expression for hair loss treatment. ACS Nano. 2026;20(11):9459–9481. 41821498 10.1021/acsnano.5c21849

[17] Lei M, Yang L, Chuong CM. Getting to the core of the dermal papilla. J Invest Dermatol. 2017;137(11):2250–2253. 29055410 10.1016/j.jid.2017.07.824

[18] Lamouille S, Xu J, Derynck R. Molecular mechanisms of epithelial-mesenchymal transition. Nat Rev Mol Cell Biol. 2014;15(3):178–196. 24556840 10.1038/nrm3758 PMC4240281

[19] Zhang CX, Huang RY, Sheng G, Thiery JP. Epithelial-mesenchymal transition. Cell. 2025;188(20):5436–5486. 41043405 10.1016/j.cell.2025.08.033

[20] Le Guen L, Marchal S, Faure S, de Santa Barbara P. Mesenchymal-epithelial interactions during digestive tract development and epithelial stem cell regeneration. Cell Mol Life Sci. 2015;72(20):3883–3896. 26126787 10.1007/s00018-015-1975-2 PMC5395663

[21] Cunha GR, Donjacour AA. Mesenchymal-epithelial interactions in the growth and development of the prostate. Cancer Treat Res. 1989;46:159–175. 2577188 10.1007/978-1-4613-1595-7_9

[22] Yu T, Klein OD. Molecular and cellular mechanisms of tooth development, homeostasis and repair. Development. 2020;147(2). 10.1242/dev.184754 PMC6983727 31980484

[23] Pleniceanu O, Harari-Steinberg O, Dekel B. Concise review: Kidney stem/progenitor cells: Differentiate, sort out, or reprogram? Stem Cells. 2010;28(9):1649–1660. 20652959 10.1002/stem.486 PMC2996087

[24] Saito A, Horie M, Nagase T. TGF-β signaling in lung health and disease. Int J Mol Sci. 2018;19(8):2460. 30127261 10.3390/ijms19082460 PMC6121238

[25] Wang M, Zhou X, Zhou S, Wang M, Jiang J, Wu W, Liu T, Xu W, Zhang J, Liu D, et al. Mechanical force drives the initial mesenchymal-epithelial interaction during skin organoid development. Theranostics. 2023;13(9):2930–2945. 37284452 10.7150/thno.83217 PMC10240816

[26] Shyer AE, Rodrigues AR, Schroeder GG, Kassianidou E, Kumar S, Harland RM. Emergent cellular self-organization and mechanosensation initiate follicle pattern in the avian skin. Science. 2017;357(6353):811–815. 28705989 10.1126/science.aai7868 PMC5605277

[27] Steele L, Olabi B, Roberts K, Mazin PV, Koplev S, Tudor C, Rumney B, Admane C, Jiang T, Correa-Gallegos D, et al. A single-cell and spatial genomics atlas of human skin fibroblasts reveals shared disease-related fibroblast subtypes across tissues. Nat Immunol. 2025;26(10):1807–1820. 40993240 10.1038/s41590-025-02267-8 PMC12479362

[28] Ascensión AM, Fuertes-Alvarez S, Ibanez-Sole O, Izeta A, Araúzo-Bravo MJ. Human dermal fibroblast subpopulations are conserved across single-cell RNA sequencing studies. J Invest Dermatol. 2021;141(7):1735–1744.e35. 33385399 10.1016/j.jid.2020.11.028

[29] Wang C, Entwistle J, Hou G, Li Q, Turley EA. The characterization of a human RHAMM cDNA: Conservation of the hyaluronan-binding domains. Gene. 1996;174(2):299–306. 8890751 10.1016/0378-1119(96)00080-7

[30] Kalmyrzaev B, Pharoah PD, Easton DF, Ponder BA, Dunning AM, SEARCH Team. Hyaluronan-mediated motility receptor gene single nucleotide polymorphisms and risk of breast cancer. Cancer Epidemiol Biomarkers Prev. 2008;17(12):3618–3620. 19064580 10.1158/1055-9965.EPI-08-0216

[31] Song JM, Molla K, Anandharaj A, Cornax I, MG OS, Kirtane AR, Panyam J, Kassie F. Triptolide suppresses the in vitro and in vivo growth of lung cancer cells by targeting hyaluronan-CD44/RHAMM signaling. Oncotarget. 2017;8(16):26927–26940. 28460475 10.18632/oncotarget.15879 PMC5432308

[32] Li KN, Chovatiya G, Ko DY, Sureshbabu S, Tumbar T. Blood endothelial ALK1-BMP4 signaling axis regulates adult hair follicle stem cell activation. EMBO J. 2023;42(10): Article e112196. 36994549 10.15252/embj.2022112196 PMC10183823

[33] Li KN, Jain P, He CH, Eun FC, Kang S, Tumbar T. Skin vasculature and hair follicle cross-talking associated with stem cell activation and tissue homeostasis. Elife. 2019;8: Article e45977. 31343406 10.7554/eLife.45977 PMC6684267

[34] Shang Y, Li M, Zhang L, Han C, Shen K, Wang K, Li Y, Zhang Y, Luo L, Jia Y, et al. Exosomes derived from mouse vibrissa dermal papilla cells promote hair follicle regeneration during wound healing by activating Wnt/β-catenin signaling pathway. J Nanobiotechnol. 2024;22(1):425. 10.1186/s12951-024-02689-w PMC11264511 39030543

[35] Xu Z, Wang W, Jiang K, Yu Z, Huang H, Wang F, Zhou B, Chen T. Embryonic attenuated Wnt/β-catenin signaling defines niche location and long-term stem cell fate in hair follicle. Elife. 2015;4: Article e10567. 26653852 10.7554/eLife.10567 PMC4758985

[36] Hong ZX, Zhu ST, Li H, Luo JZ, Yang Y, An Y, Wang X, Wang K. Bioengineered skin organoids: From development to applications. Mil Med Res. 2023;10(1):40. 37605220 10.1186/s40779-023-00475-7 PMC10463602

[37] Lei M, Schumacher LJ, Lai YC, Juan WT, Yeh CY, Wu P, Jiang TX, Baker RE, Widelitz RB, Yang L, et al. Self-organization process in newborn skin organoid formation inspires strategy to restore hair regeneration of adult cells. Proc Natl Acad Sci USA. 2017;114(34):E7101–e7110. 28798065 10.1073/pnas.1700475114 PMC5576784

[38] Lei M, Jiang J, Wang M, Wu W, Zhang J, Liu W, Zhou W, Lai YC, Jiang TX, Widelitz RB, et al. Epidermal-dermal coupled spheroids are important for tissue pattern regeneration in reconstituted skin explant cultures. NPJ Regener Med. 2023;8(1):65. 10.1038/s41536-023-00340-0 PMC10667216 37996466

[39] Sproul EP, Argraves WS. A cytokine axis regulates elastin formation and degradation. Matrix Biol. 2013;32(2):86–94. 23160093 10.1016/j.matbio.2012.11.004 PMC3633528

[40] He X, Zhang B, Qi R, Lei M, Xu X. Research advances in purification, expression, and function of fibroblast growth factor 9. Regen Repair Rehabil. 2025;1(3):64–69.

[41] Shin JW, Kwon SH, Choi JY, Na JI, Huh CH, Choi HR, Park KC. Molecular mechanisms of dermal aging and antiaging approaches. Int J Mol Sci. 2019;20(9):2126. 31036793 10.3390/ijms20092126 PMC6540032

[42] Zhang J, Yu H, Man MQ, Hu L. Aging in the dermis: Fibroblast senescence and its significance. Aging Cell. 2024;23(2): Article e14054. 38040661 10.1111/acel.14054 PMC10861215

[43] Hynes RO. The extracellular matrix: Not just pretty fibrils. Science. 2009;326(5957):1216–1219. 19965464 10.1126/science.1176009 PMC3536535

[44] Lu P, Takai K, Weaver VM, Werb Z. Extracellular matrix degradation and remodeling in development and disease. Cold Spring Harb Perspect Biol. 2011;3(12): Article a005058. 21917992 10.1101/cshperspect.a005058 PMC3225943

[45] Chen CY, Xu R, Mo M, Wu J, Chi J, Wu ZR, Wang Y, Zhong XC, Lin XY, Liu Y, et al. Transcriptome-guided development of a fibrosis-reversal compound reduces skin scarring and allows regeneration via mitochondrial uncoupling. Cell Rep Med. 2026;7(5): Article 102821. 42155361 10.1016/j.xcrm.2026.102821 PMC13198262

[46] Tao Y, Yang Q, Wang L, Zhang J, Zhu X, Sun Q, Han Y, Luo Q, Wang Y, Guo X, et al. β-Catenin activation in hair follicle dermal stem cells induces ectopic hair outgrowth and skin fibrosis. J Mol Cell Biol. 2019;11(1):26–38. 29771334 10.1093/jmcb/mjy032

[47] Missinato MA, Tobita K, Romano N, Carroll JA, Tsang M. Extracellular component hyaluronic acid and its receptor Hmmr are required for epicardial EMT during heart regeneration. Cardiovasc Res. 2015;107(4):487–498. 26156497 10.1093/cvr/cvv190 PMC4540147

[48] Saraswathibhatla A, Indana D, Chaudhuri O. Cell-extracellular matrix mechanotransduction in 3D. Nat Rev Mol Cell Biol. 2023;24(7):495–516. 36849594 10.1038/s41580-023-00583-1 PMC10656994

[49] Zhu Y, Chen J, Chen C, Tang R, Xu J, Shi S, Yu X. Deciphering mechanical cues in the microenvironment: From non-malignant settings to tumor progression. Biomark Res. 2025;13(1):11. 39849659 10.1186/s40364-025-00727-9 PMC11755887

[50] Humphrey JD, Dufresne ER, Schwartz MA. Mechanotransduction and extracellular matrix homeostasis. Nat Rev Mol Cell Biol. 2014;15(812):802–812. 25355505 10.1038/nrm3896 PMC4513363

[51] Morgan BA. The dermal papilla: An instructive niche for epithelial stem and progenitor cells in development and regeneration of the hair follicle. Cold Spring Harb Perspect Med. 2014;4(7): Article a015180. 24985131 10.1101/cshperspect.a015180 PMC4066645

