# Supplementary material for: Hmmr+ Fibroblasts Facilitate Hair Regeneration by Biomechanical Sensing of Extracellular Matrix Viscoelasticity
Source: Research (Wash D C). 2026 Sep 14;9:1430. doi: 10.34133/research.1430 (PMC13572799; doi:10.34133/research.1430)
Supplement: Supplementary file 1 [file research.1430.f1.pdf]

Fig. S1.

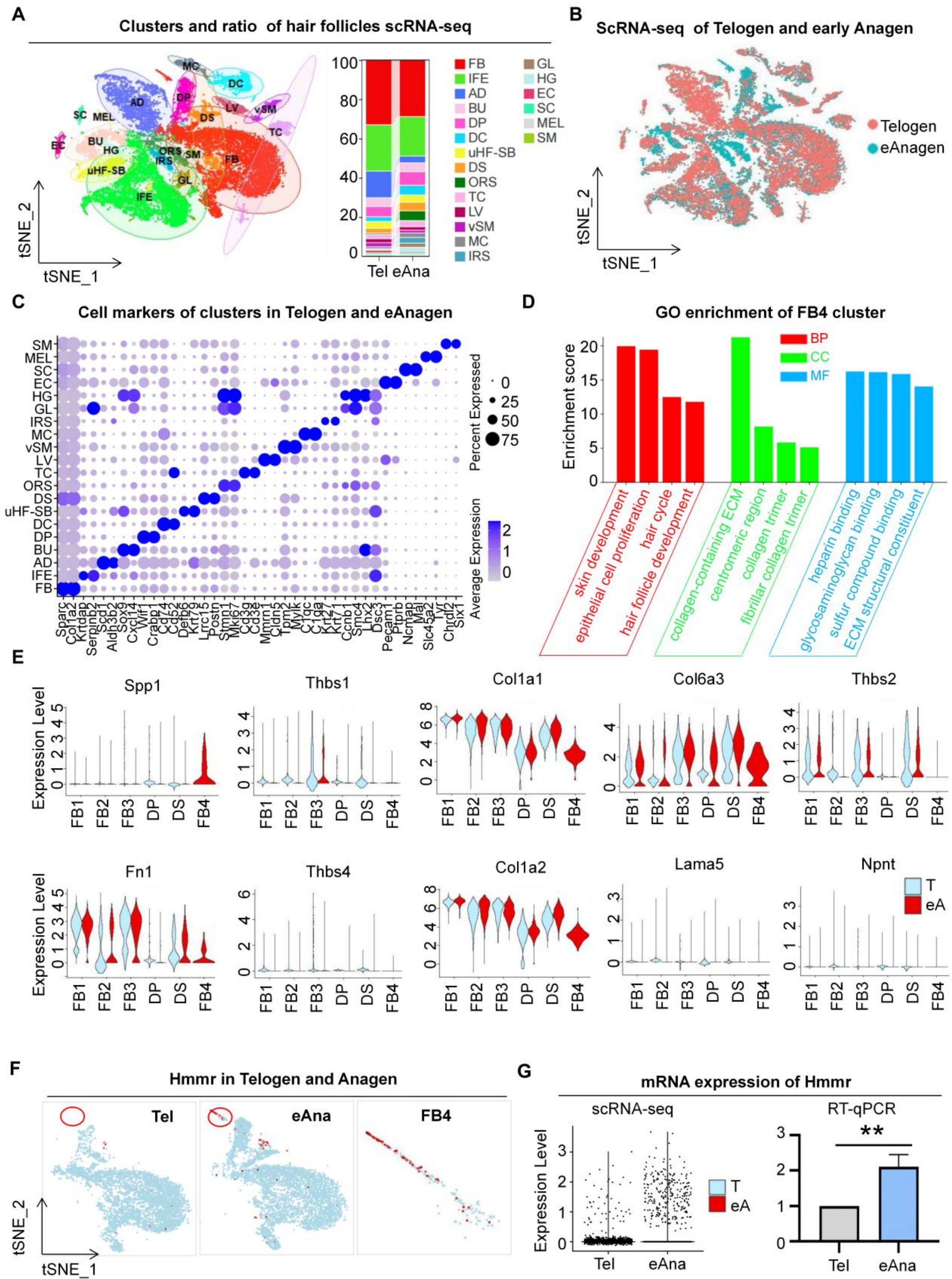
A
Clusters and ratio of hair follicles scRNA-seq
B
ScRNA-seq of Telogen and early Anagen
Telogen
eAnagen
C
Cell markers of clusters in Telogen and eAnagen
D
GO enrichment of FB4 cluster
BP
CC
MF
Enrichment score
skin development
epithelial cell proliferation
hair cycle
hair follicle development
collagen-containing ECM
centromeric region
collagen trimer
fibrillar collagen trimer
heparin binding
glycosaminoglycan binding
sulfur compound binding
ECM structural constituent
E
Spp1
Thbs1
Col1a1
Col6a3
Thbs2
Fn1
Thbs4
Col1a2
Lama5
Npnt
Expression Level
F
Hmmr in Telogen and Anagen
Tel
eAna
FB4
G
mRNA expression of Hmmr
scRNA-seq
RT-qPCR

Fig. S1. Hmmr+ fibroblast subset emergence: spatiotemporal dynamics in telogen-to-anagen transition. (A) Single-cell clustering analysis comparing telogen and early anagen hair follicle samples, and percentage of each cell subpopulation between the two stages. (B) tSNE plot showing clustering of scRNA-seq data from mouse skin hair follicles at telogen and early anagen phases. (C) Bubble plot showing marker genes for each cell subpopulation. (D) GO enrichment analysis revealing functions of the FB4 subpopulation. (E) VlnPlot displaying expression of other genes involved in the ECM-receptor interaction pathway. (F) Feature plots showing differential expression of Hmmr between telogen and early anagen hair follicles in scRNA-seq data. (G) qRT-PCR validation of Hmmr expression differences between telogen and early anagen hair follicles (n = 3; $p < 0.05$, **$p < 0.01$, ***$p < 0.001$).

.

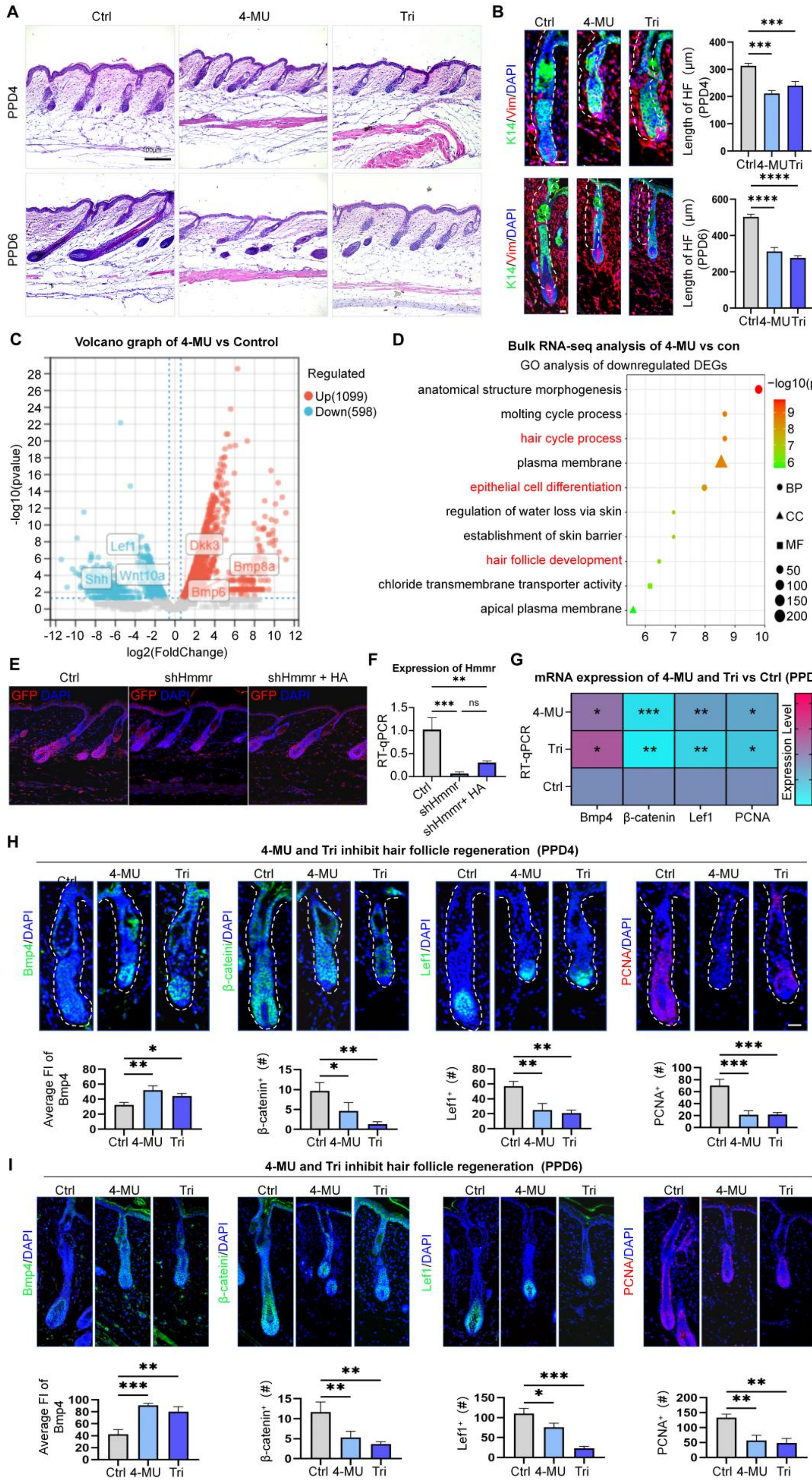

A
Ctrl
4-MU
Tri
PPD4
PPD6
B
K14/Vim/DAPI
Length of HF (μm) (PPD4)
Length of HF (μm) (PPD6)
C
Volcano graph of 4-MU vs Control
Regulated
Up(1099)
Down(598)
-log10(pvalue)
log2(FoldChange)
Lef1
Shh
Wnt10a
Dkk3
Bmp8a
Bmp6
D
Bulk RNA-seq analysis of 4-MU vs con
GO analysis of downregulated DEGs
anatomical structure morphogenesis
molting cycle process
hair cycle process
plasma membrane
epithelial cell differentiation
regulation of water loss via skin
establishment of skin barrier
hair follicle development
chloride transmembrane transporter activity
apical plasma membrane
−log10(p)
BP
CC
MF
E
shHmmr
shHmmr + HA
GFP DAPI
F
Expression of Hmmr
RT-qPCR
G
mRNA expression of 4-MU and Tri vs Ctrl (PPD4)
Expression Level
Bmp4
β-catenin
Lef1
PCNA
H
4-MU and Tri inhibit hair follicle regeneration (PPD4)
Bmp4/DAPI
β-cateini/DAPI
Lef1/DAPI
PCNA/DAPI
Average FI of Bmp4
β-catenin⁺ (#)
Lef1⁺ (#)
PCNA⁺ (#)
I
4-MU and Tri inhibit hair follicle regeneration (PPD6)

Fig. S2. HA-Hmmr signaling drives hair regeneration. (A) HE staining showing the effects of subcutaneous 4-MU and Triptolide injections on hair follicle regeneration. Scale bar, 100 µm. (B) IF staining of Keratin 14 (K14) and Vimentin (Vim) demonstrating the effects of 4-MU and Triptolide on hair follicle length and morphology, with quantification of hair follicle length. Scare bar, 25 µm. (C) Volcano plot displaying upregulated and downregulated genes between the 4-MU treatment group and the control group. (D) GO enrichment analysis of bulk RNA-seq data showing downregulated genes in dermal fibroblasts after 4-Methylumbelliferone (4-MU)-mediated inhibition of Hmmr function. (E) IF staining showing GFP expression following subcutaneous injection of adenovirus for Hmmr knockdown. (F) RT-qPCR analysis showing the relative Hmmr expression following subcutaneous adenoviral-mediated Hmmr knockdown. (G) RT-qPCR analysis of the effects of 4-MU and Triptolide (Hmmr inhibitors) on the expression of Bmp4, β-catenin, Lef1, and PCNA. (H) IF staining showing the expression of Bmp4, β-catenin, Lef1, and PCNA after 4-MU and Triptolide treatment (PPD4). Scale bar,25 µm (I) IF staining showing the expression of Bmp4, β-catenin, Lef1, and PCNA after 4-MU and Triptolide treatment (PPD6). Scale bar,50 µm (n = 3; $^*p < 0.05$, $^{**}p < 0.01$, $^{***}p < 0.001$).

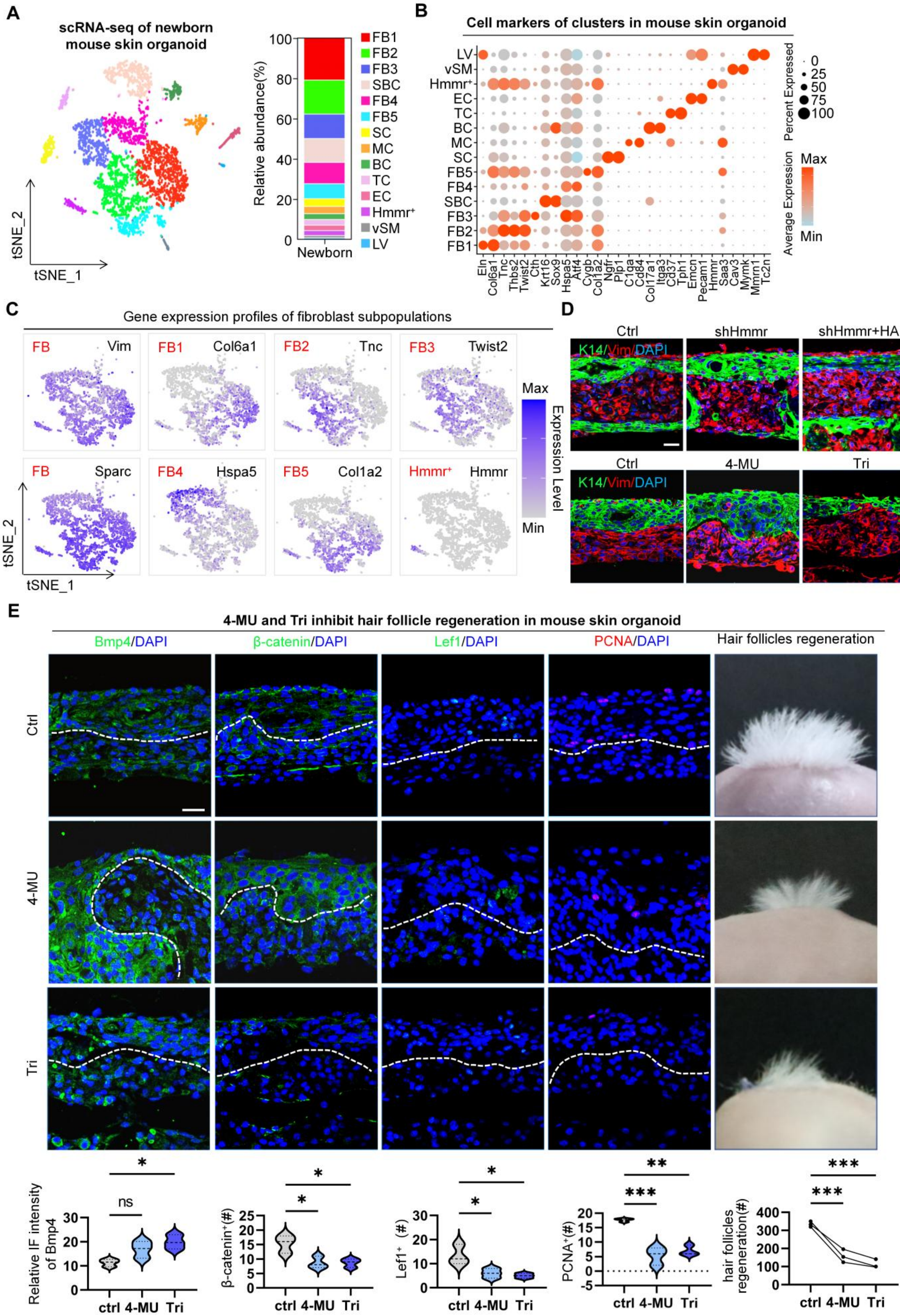

A
scRNA-seq of newborn mouse skin organoid
tSNE_1
tSNE_2
Relative abundance(%)
Newborn
FB1
FB2
FB3
SBC
FB4
FB5
SC
MC
BC
TC
EC
Hmmr⁺
vSM
LV
B
Cell markers of clusters in mouse skin organoid
Percent Expressed
Average Expression
Max
Min
Eln
Col6a1
Tnc
Thbs2
Twist2
Cth
Krt16
Sox9
Hspa5
Atf4
Cygb
Col1a2
Ngfr
Plp1
C1qa
Cd84
Col17a1
Itga3
Cd37
Tph1
Emcn
Pecam1
Hmmr
Saa3
Cav3
Mymk
Mmrn1
Tc2n
C
Gene expression profiles of fibroblast subpopulations
FB Vim
FB1 Col6a1
FB2 Tnc
FB3 Twist2
FB Sparc
FB4 Hspa5
FB5 Col1a2
Hmmr⁺ Hmmr
Expression Level
D
Ctrl
shHmmr
shHmmr+HA
K14/Vim/DAPI
4-MU
Tri
E
4-MU and Tri inhibit hair follicle regeneration in mouse skin organoid
Bmp4/DAPI
β-catenin/DAPI
Lef1/DAPI
PCNA/DAPI
Hair follicles regeneration
Ctrl
4-MU
Tri
Relative IF intensity of Bmp4
β-catenin⁺(#)
Lef1⁺ (#)
PCNA⁺(#)
hair follicles regeneration(#)
ctrl 4-MU Tri
ns

Fig. S3. Functional HMMR-high dermal niche enables hair follicle neogenesis in organoids. (A) Single-cell clustering and cell proportion plot of mouse skin organoid. (B) Bubble plot showing marker genes for each cell subpopulation. (C) FeaturePlot showing marker genes of various dermal cell subpopulations. (D) IF staining showing morphological changes in mouse skin organoids following 4-MU treatment, Triptolide intervention, and adenoviral-mediated Hmmr knockdown. Scale bar, 25 μm. (E) Left: IF staining showing the effects of 4-MU and Triptolide (Hmmr inhibitors) on the expression of Bmp4, β-catenin, Lef1, and PCNA in the mouse skin organoids; Right: Quantitative comparison of hair regeneration after transplantation into nude mice between 4-MU/Triptolide treatment groups and the control group. Scale bar, 25 μm. (n = 3; $p < 0.05$, $^{**}p < 0.01$, $^{***}p < 0.001$).

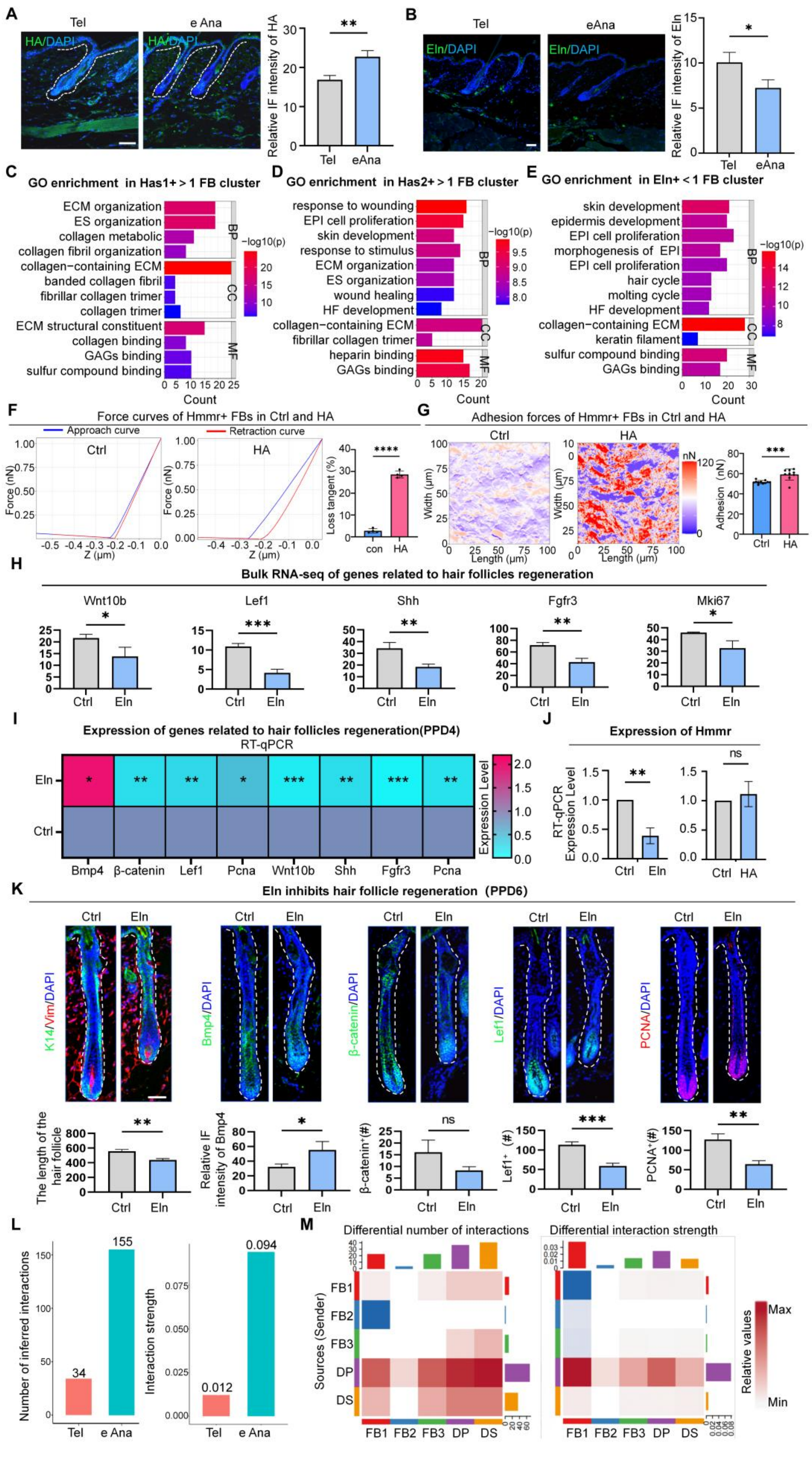

A
Tel
e Ana
HA/DAPI
Relative IF intensity of HA
**
Tel
eAna
B
Tel
eAna
Eln/DAPI
Relative IF intensity of Eln
*
Tel
eAna
C
GO enrichment in Has1+ > 1 FB cluster
ECM organization
ES organization
collagen metabolic
collagen fibril organization
collagen−containing ECM
banded collagen fibril
fibrillar collagen trimer
collagen trimer
ECM structural constituent
collagen binding
GAGs binding
sulfur compound binding
BP
CC
MF
−log10(p)
Count
D
GO enrichment in Has2+ > 1 FB cluster
response to wounding
EPI cell proliferation
skin development
response to stimulus
ECM organization
ES organization
wound healing
HF development
collagen−containing ECM
fibrillar collagen trimer
heparin binding
GAGs binding
BP
CC
MF
−log10(p)
Count
E
GO enrichment in Eln+ < 1 FB cluster
skin development
epidermis development
EPI cell proliferation
morphogenesis of EPI
EPI cell proliferation
hair cycle
molting cycle
HF development
collagen−containing ECM
keratin filament
sulfur compound binding
GAGs binding
BP
CC
MF
−log10(p)
Count
F
Force curves of Hmmr+ FBs in Ctrl and HA
Approach curve
Retraction curve
Ctrl
HA
Force (nN)
Z (μm)
Loss tangent (%)
****
con
HA
G
Adhesion forces of Hmmr+ FBs in Ctrl and HA
Ctrl
HA
Width (μm)
Length (μm)
nN
Adhesion (nN)
***
Ctrl
HA
H
Bulk RNA-seq of genes related to hair follicles regeneration
Wnt10b
Lef1
Shh
Fgfr3
Mki67
Ctrl
Eln
I
Expression of genes related to hair follicles regeneration(PPD4)
RT-qPCR
Eln
Ctrl
Bmp4
β-catenin
Lef1
Pcna
Wnt10b
Shh
Fgfr3
Pcna
Expression Level
J
Expression of Hmmr
RT-qPCR
Expression Level
**
ns
Ctrl
Eln
Ctrl
HA
K
Eln inhibits hair follicle regeneration (PPD6)
Ctrl
Eln
K14/Vim/DAPI
Bmp4/DAPI
β-catenin/DAPI
Lef1/DAPI
PCNA/DAPI
The length of the hair follicle
Relative IF intensity of Bmp4
β-catenin⁺(#)
Lef1⁺ (#)
PCNA⁺(#)
L
Number of inferred interactions
34
155
Tel
e Ana
Interaction strength
0.012
0.094
Tel
e Ana
M
Differential number of interactions
Differential interaction strength
Sources (Sender)
FB1
FB2
FB3
DP
DS
Relative values
Max
Min

Fig. S4. Mechanical crosstalk between fibroblasts and ECM directs follicle regeneration. (A-B) IF staining showing differential expression of HA and Eln in telogen and early anagen hair follicles. Scale bar, 50 µm. (C-E) GO enrichment analysis revealing biological functions of the upregulated genes in Hyaluronan Synthase 1/2 (Has1/Has2)-positive cells and the downregulated genes in Elastin (Eln)-positive cells. (F) Representative loading – unloading curves and corresponding loss coefficients of ECM in HMMR$^+$ fibroblast regions following subcutaneous HA treatment detected by AFM. (G) AFM-based quantitative analysis of ECM adhesion force in HMMR$^+$ fibroblast regions following subcutaneous HA treatment. (H) Bulk RNA-seq showing the expression of hair follicle regeneration-related genes. (I) qRT-PCR showing the expression of hair follicle regeneration-related genes. (J) qRT-PCR showing the effect of subcutaneous Eln and HA injections on Hmmr expression. (K) IF staining highlighting the impact of subcutaneous Eln injection on the expression of Bmp4, β-catenin, Lef1, and PCNA (PPD6). Scale bar, 50 µm. (L) Cellchat analysis comparing the quantity and intensity of dermal interactions during the telogen and early anagen phases. (M) Heatmap visualizing differences in the number and strength of dermal interactions between telogen and early anagen phases.

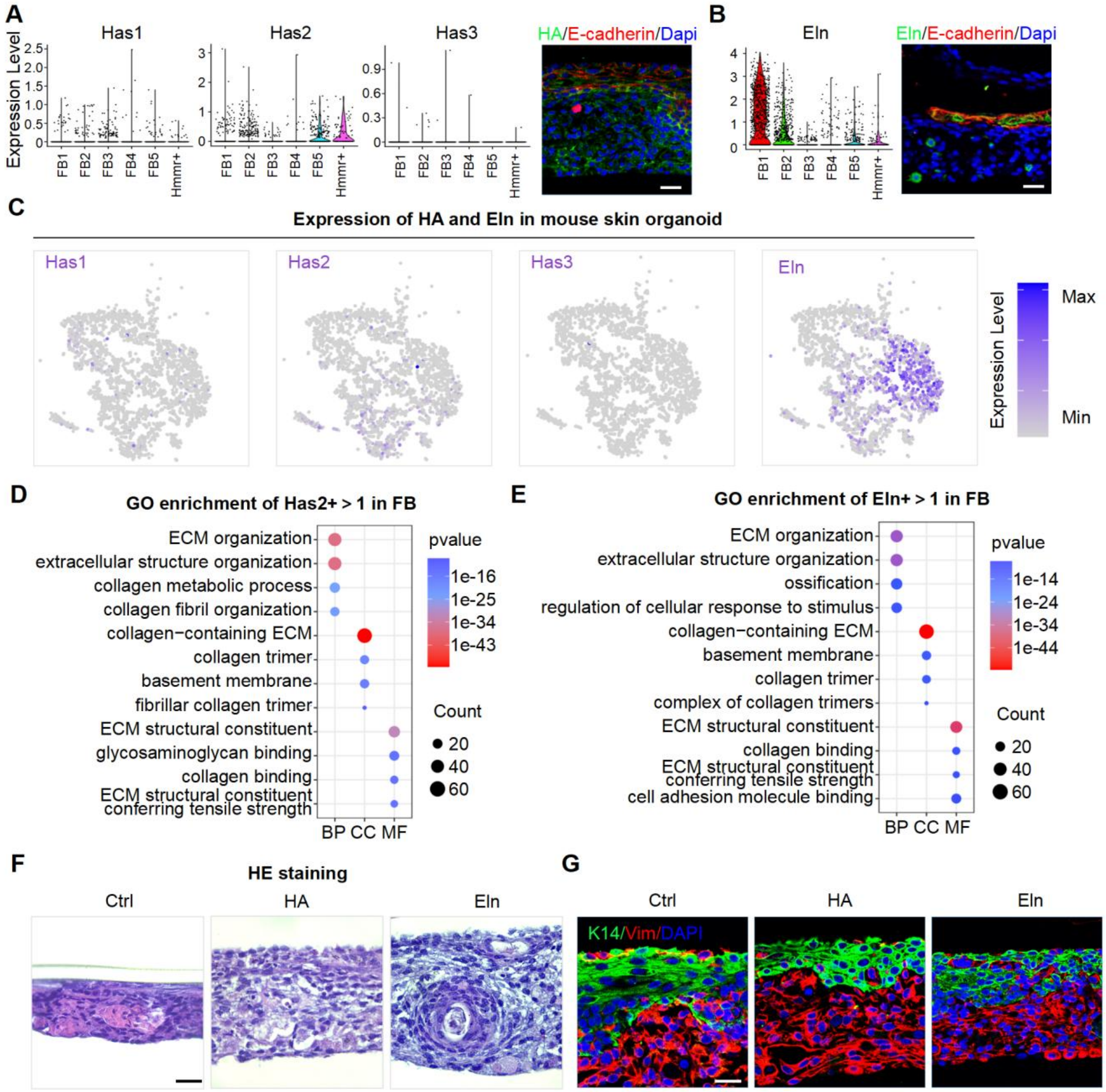

Fig. S5. Reduced ECM elasticity promotes hair regeneration in mouse skin organoids. (A) Gene expression analysis of Has1, Has2, and Hyaluronan Synthase 3 (Has3); Left: VlnPlot showing expression distribution of Has1, Has2, and Has3; Right: IF staining revealing spatial localization of HA in mouse skin organoids. Scale bar, 25 μm. (B) Expression analysis of Eln; Left: VlnPlot displaying Eln expression in mouse skin organoids; Right: IF staining showing spatial distribution of elastin in mouse skin organoids. Scale bar, 25 μm. (C) Feature plots visualizing spatial expression patterns of Has1, Has2, Has3, and Eln in dermal fibroblasts of mouse skin organoids. (D-E) GO enrichment analysis revealing biological functions of genes in Has2^{+} and Eln^{+} fibroblasts. (F-G) HE staining showing morphological changes and IF staining demonstrating protein expression in mouse skin organoids following subcutaneous injection of HA and Eln. Scale bar, 25 μm.

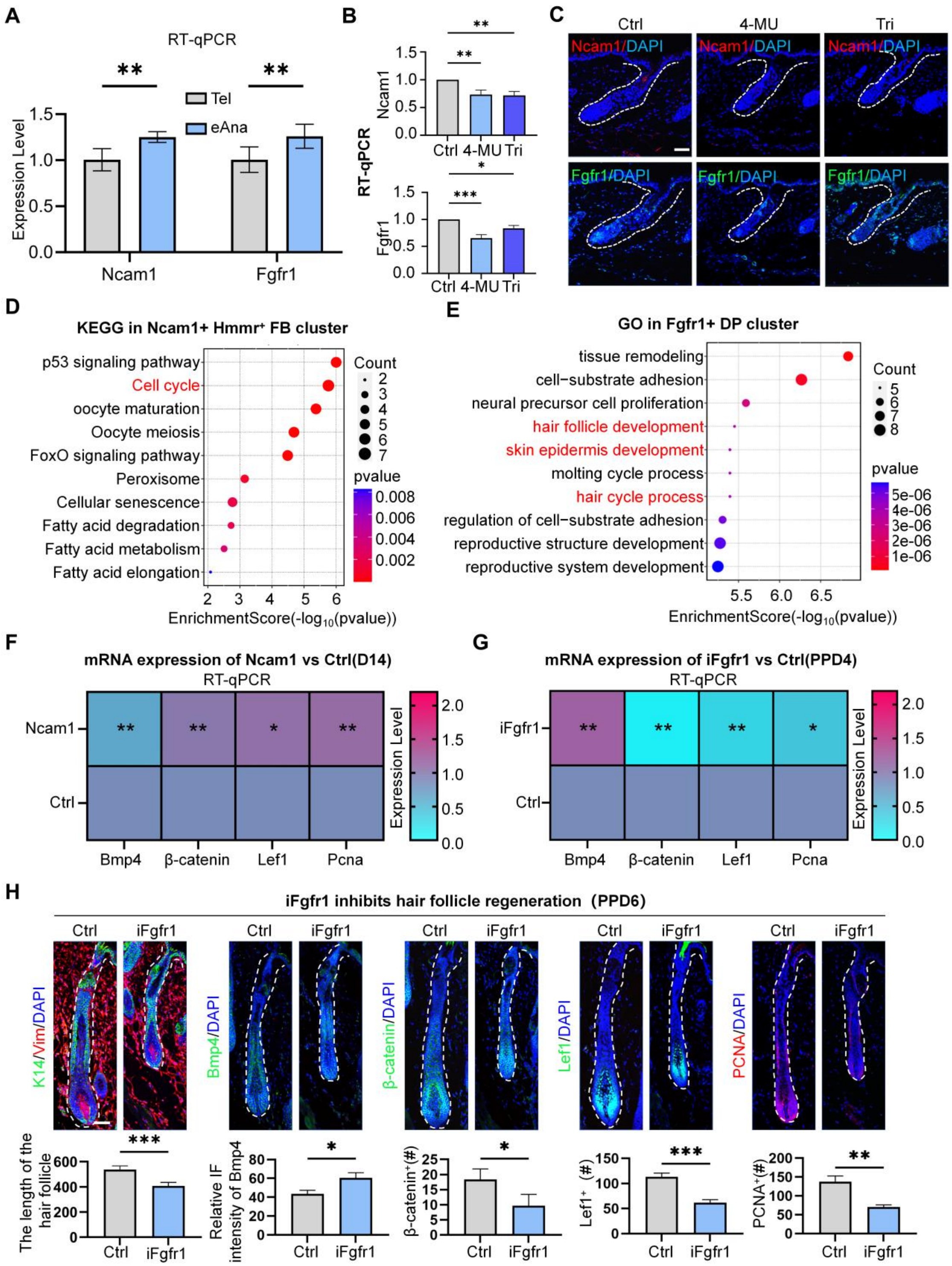

Fig. S6. Hmmr$^+$ FB interacts with DP by the Ncam1-Fgfr1 pathway to promote hair follicle regeneration. (A) RT-qPCR results showing differential expression of Ncam1 and Fgfr1 between telogen and early anagen hair follicle. (B-C) RT-qPCR analysis and IF staining demonstrating the effects of 4-MU and Triptolide (Hmmr inhibitors) on the expression of Ncam1

and Fgfr1 in dermal fibroblasts. Scale bar, 50 μm. (D) KEGG enrichment analysis revealing functional pathways enriched in Ncam1-positive cells within $Hmmr^{+}$ FBs. (E) GO enrichment analysis showing biological functions of Fgfr1-positive cells in DP. (F) RT-qPCR results showing the effects of subcutaneous injection of Ncam1 on the expression of Bmp4, β-catenin, Lef1, and PCNA on day 14 post-Ncam1 injection (D14). (G) qRT-PCR results showing the effects of subcutaneous injection of PD173074 (a selective Fgfr1 inhibitor) on the expression of Bmp4, β-catenin, Lef1, and PCNA (PPD4). (H) IF staining showing the effects of subcutaneous injection of PD173074 on the expression of Bmp4, β-catenin, Lef1, and PCNA in hair follicles (PPD6). Scale bar, 50 μm. ($n = 3$; $*p < 0.05$, $**p < 0.01$, $***p < 0.001$).

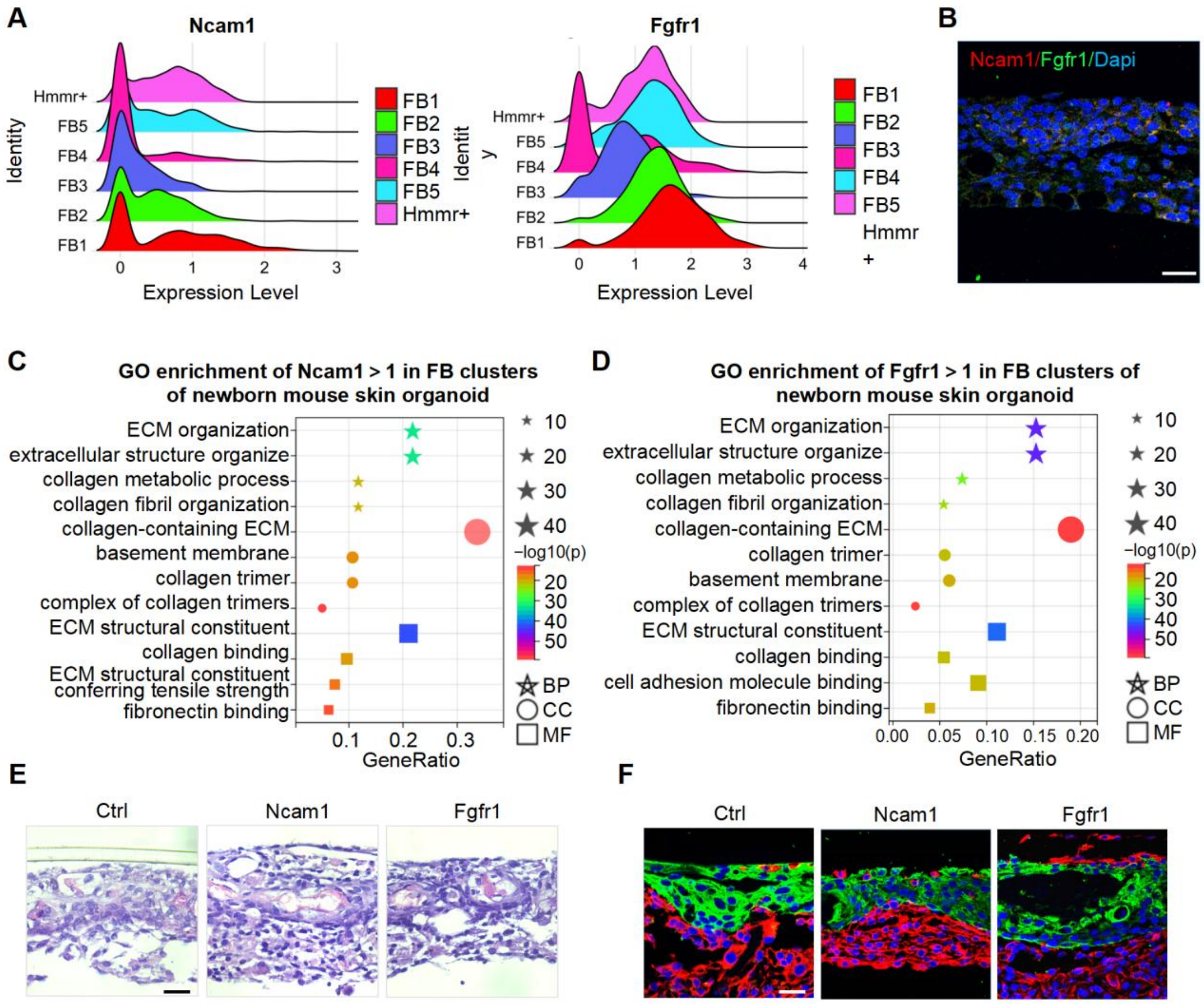

Fig. S7. Hmmr$^+$ FB interacts with DP via the Ncam1-Fgfr1 pathway to promote hair follicle regeneration. (A-B) Mountain plots and IF staining demonstrating the expression of Ncam1 and Fgfr1 in dermal fibroblasts. Scale bar, 25 μm. (C-D) GO enrichment analysis revealing biological functions of Ncam1$^+$ cells and Fgfr1$^+$ cells in dermal cells. (E-F) HE staining demonstrating morphological changes and IF staining demonstrating protein expression in hair follicles within mouse skin organoids after treatment with Ncam1 and the Fgfr1 selective inhibitor PD173074. Scale bar, 25 μm.

Primer list:

Gene	PrimerBank ID		Sequence (5' -> 3')	Length	Tm	Location
Ctnnb1	6671684a1	Forward Primer	ATGGAGCCGGACAGAAAAGC	20	62.5	40-59
		Reverse Primer	CTTGCCACTCAGGGAAGGA	19	60.9	147-129
Bmp4	6680796a1	Forward Primer	TTCCTGGTAACCGAATGCTGA	21	61.1	5-25
		Reverse Primer	CCTGAATCTCGGCGACTTTTT	21	60.6	118-98
Lef1	27735019a1	Forward Primer	TGTTTATCCCATCACGGGTGG	21	61.8	645-665
		Reverse Primer	CATGGAAGTGTCGCCTGACAG	21	63	711-691
Pcna	7242171a1	Forward Primer	TTTGAGGCACGCCTGATCC	19	62.3	4-22
		Reverse Primer	GGAGACGTGAGACGAGTCCAT	21	63	138-118
Ncam1	817988a1	Forward Primer	ACCACCGTCACCACTAACTCT	21	62.5	2476-2496
		Reverse Primer	TGGGGCAATACTGGAGGTCA	20	62.5	2577-2558
Fgfr1	26390450a1	Forward Primer	TAATACCACCGACAAGGAAATGG	23	60	681-703
		Reverse Primer	TGATGGGAGAGTCCGATAGAGT	22	61.2	788-767
Has1	6680169a1	Forward Primer	GGCGAGCACTCACGATCATC	20	63	59-78
		Reverse Primer	AGGAGTCCATAGCGATCTGAAG	22	60.8	158-137
Has2	6996921a1	Forward Primer	TGTGAGAGGTTTCTATGTGTCCT	23	60.4	7-29
		Reverse Primer	ACCGTACAGTCCAAATGAGAAGT	23	61.2	150-128
Has3	6680173a1	Forward Primer	GTGGGCACCAGTCTGTTTG	19	60.9	34-52
		Reverse Primer	CCACTGAACGCGACCTCTG	19	62.6	265-247
Eln	31542606a1	Forward Primer	TTGCTGATCCTCTTGCTCAAC	21	60.3	40-60
		Reverse Primer	GCCCCTGGATAATAGACTCCAC	22	61.4	152-131
Hmmr	7305145a1	Forward Primer	CCTTGCTTGCTTCGGCTAAAA	21	61.5	182-202
		Reverse Primer	CTGCTGCATTGAGCTTTGCT	20	61.6	370-351
Wnt10 b	1546017a1	Forward Primer	GCGGGTCTCCTGTTCTTGG	19	62.3	43-61
		Reverse Primer	CCGGGAAGTTTAAGGCCCAG	20	62.5	113-94
Fgfr3	2558918a1	Forward Primer	GCCTGCGTGCTAGTGTTCT	19	62.3	13-31
		Reverse Primer	TACCATCCTTAGCCCAGACCG	21	62.8	229-209
Mki67	1177528a1	Forward Primer	ATCATTGACCGCTCCTTTAGGT	22	61.2	268-289
		Reverse Primer	GCTCGCCTTGATGGTTCCT	19	62	371-353
